# Estimating the impact of structural directionality: How reliable are undirected connectomes?

**DOI:** 10.1162/netn_a_00040

**Published:** 2018-06-01

**Authors:** Penelope Kale, Andrew Zalesky, Leonardo L. Gollo

**Affiliations:** QIMR Berghofer Medical Research Institute, Australia; University of Queensland, Australia; Melbourne Neuropsychiatry Centre and Department of Biomedical Engineering, University of Melbourne, Australia; QIMR Berghofer Medical Research Institute, Australia; University of Queensland, Australia

**Keywords:** Directionality, Connectome, Structural connectivity, Graph theory, Hubs, False positives

## Abstract

Directionality is a fundamental feature of network connections. Most structural brain networks are intrinsically directed because of the nature of chemical synapses, which comprise most neuronal connections. Because of the limitations of noninvasive imaging techniques, the directionality of connections between structurally connected regions of the human brain cannot be confirmed. Hence, connections are represented as undirected, and it is still unknown how this lack of directionality affects brain network topology. Using six directed brain networks from different species and parcellations (cat, mouse, *C. elegans*, and three macaque networks), we estimate the inaccuracies in network measures (degree, betweenness, clustering coefficient, path length, global efficiency, participation index, and small-worldness) associated with the removal of the directionality of connections. We employ three different methods to render directed brain networks undirected: (a) remove unidirectional connections, (b) add reciprocal connections, and (c) combine equal numbers of removed and added unidirectional connections. We quantify the extent of inaccuracy in network measures introduced through neglecting connection directionality for individual nodes and across the network. We find that the coarse division between core and peripheral nodes remains accurate for undirected networks. However, hub nodes differ considerably when directionality is neglected. Comparing the different methods to generate undirected networks from directed ones, we generally find that the addition of reciprocal connections (*false positives*) causes larger errors in graph-theoretic measures than the removal of the same number of directed connections (*false negatives*). These findings suggest that directionality plays an essential role in shaping brain networks and highlight some limitations of undirected connectomes.

## Author Summary

Most brain networks are inherently directed because of the nature of chemical synapses, which comprise most neuronal connections. However, owing to limitations of non-invasive techniques, the human connectome is represented without directionality. To better understand the effects of directionality on graph-theoretic measures of anatomical networks, we compare brain networks across species when directionality is neglected, and find that hubs become vulnerable. Moreover, the addition of few false-positive reciprocal connections is more detrimental to network measures than the removal of the same number of directed false-negative connections. Our results suggest that directionality plays an essential role in shaping brain networks and highlight some limitations of undirected connectomes.

## INTRODUCTION

Connectomes provide a comprehensive network description of structural brain connectivity (Sporns, Tononi, & Kötter, 2005). Large-scale connectomes mapped in humans are typically represented and analyzed as undirected networks, because of the inability of noninvasive connectome mapping techniques to resolve the directionality (afferent or efferent) of white matter fibers. Reducing an inherently directed network such as the connectome to an undirected network is a simplification that may introduce inaccuracies in graph-theoretic analyses. For example, the flow of action potentials along an axon is mostly only ever in one direction, and thus analyses of information flow are critically dependent on connection directionality. This study aims to systematically and comprehensively characterize the impact of representing and analyzing connectomes as undirected networks.

At the neuronal level, the connections between nodes (neurons) are given by synapses, and the great majority of them are chemical, which have distinctive pre- and postsynaptic terminals determining the direction of neurotransmitter flux (Kandel, Schwartz, Jessell, Siegelbaum, & Hudspeth, 2000). This structural feature of chemical synapses emphasizes the importance of directionality for the connections, and therefore for the whole network. Invasive techniques to map connectomes such as tract tracing (Kötter, 2004; Dong, 2008; Scannell, Burns, Hilgetag, O’Neil, & Young, 1999; Sporns, Honey, & Kötter, 2007) or electron microscopy (Achacoso & Yamamoto, 1992; White, Southgate, Thomson, & Brenner, 1986) can detect the directionality of the connections. Conversely, human connectomes are currently mapped with noninvasive tractography methods performed on diffusion-weighted magnetic resonance imaging data (Assaf & Basser, 2005; Hagmann et al., 2008; Tournier, Calamante, & Connelly, 2012). While methods for improving the quality of diffusion-based connectomes have advanced in recent years, and numerous tractography algorithms have been developed to reconstruct axonal fiber bundles, they cannot provide any information about the directionality of the connections. Therefore, analyses of the human connectome, as well as modeling studies that use the human connectivity matrix, are compromised by the lack of information regarding directionality, which is one of the most fundamental features of complex networks.

In the absence of directionality, networks are considered undirected and therefore the connections only represent the existence of a relationship between nodes. This is the case for scientific coauthorship networks (Newman, 2004), film actor networks (Watts & Strogatz, 1998), and functional networks defined by symmetric functions such as the Pearson correlation (Biswal, Zerrin Yetkin, Haughton, & Hyde, 1995) or the phase locking value (Aydore, Pantazis, & Leahy, 2013). Among others, studies of tractography-derived human brain networks have revealed a variety of important features such as hub regions (van den Heuvel & Sporns, 2013), modularity and clustering (Sporns, 2011; Sporns & Betzel, 2016), small-worldness (Bassett & Bullmore, 2006; Medaglia & Bassett, 2017), core-periphery structure (Hagmann et al., 2008), and the existence of a rich club (van den Heuvel & Sporns, 2011). These topological properties are not specific to the human brain. Comparisons across many species have recapitulated these features (Betzel & Bassett, 2016; Harriger, van den Heuvel, & Sporns, 2012; Towlson, Vértes, Ahnert, Schafer, & Bullmore, 2013; van den Heuvel, Bullmore, & Sporns, 2016). However, the topological characteristics of connectomes, as well as many other graph-theoretic measures, are affected by the directionality of connections (Rubinov & Sporns, 2010).

When directionality cannot be identified, undirected representations of connectomes are incomplete. Undirected networks inform the presence of a relationship between two brain regions. But these networks lack information about the asymmetry of this relationship. For example, if a directed network is represented as an undirected network, unidirectional connections are either present, which can be interpreted as a spurious addition of a reciprocal connection (*false positives*), or overlooked (*false negatives*). More specifically, if a unidirectional connection exists from node *u* to *v*, but not from *v* to *u*, then the undirected representation of this connection is either (a) an undirected connection between *u* and *v*, which can be construed as admitting a false positive from node *v* to *u*; or, (b) absence of an undirected connection between *u* and *v*, which can be construed as a false negative from node *u* to *v*. In either case, a potential error (false positive or false negative) is introduced to the undirected network.

Beyond the effect of directionality, connectomes also contain errors in the balance between overlooked and spurious connections owing to imprecisions in currently available mapping techniques (Calabrese, Badea, Cofer, Qi, & Johnson, 2015; Donahue et al., 2016). Although both error types impact the network topology, spurious (false positive) connections introduce inaccuracies in a few graph-theoretic measures (network clustering, efficiency, and modularity) in different connectomes that are at least twice as large as those found with the same number of overlooked (false negative) connections (Zalesky et al., 2016). This finding indicates that the importance of specificity is much greater than sensitivity for general connectivity in which false positives could be any absent connection and false negatives, any present connection. However, the impact of representing a directed connection as undirected, which, for practical purposes, is typically indistinguishable from a bidirectional connection, is currently unknown. Therefore, when directed networks are mapped with techniques that cannot infer directionality, it is important to establish what undirected representation is the most detrimental with respect to directionality: admitting spurious reciprocal connections (false positives) or overlooking unidirectional connections (false negatives).

Moreover, the effect of directionality on the identification of network hubs may also be important, as hubs play an important role for normal brain function (Mišić et al., 2015; van den Heuvel, Kahn, Goñi, & Sporns, 2012) as well as in neuropsychiatric disorders (Bassett et al., 2008; Crossley et al., 2014; Fornito, Zalesky, & Breakspear, 2015). But how are these highly connected regions affected by directionality? Does the classification of nodes into hubs still hold if directionality is taken into account? Furthermore, to what extent do graph-theoretic measures at the node level remain valid? The characterization of the human brain as an undirected network is often overlooked and requires investigation.

The aim of this study is to understand the limitations of analyzing inherently directed connectomes as undirected networks. Beginning with directed connectomes of the macaque, cat, mouse, and *Caenorhabditis elegans* (*C. elegans*), we study how seven graph-theoretic measures are affected as we progressively modify unidirectional connections, either deleting them or making them undirected. More specifically, we consider three schemes to progressively eliminate directionality information: removing unidirectional connections (creating false negatives), adding reciprocal connections to existing unidirectional connections (creating false positives), and removing one unidirectional connection for each reciprocal connection added, thus preserving the density and mean degree of the original network. We show how essential network features, such as the identification and classification of hubs, are affected by perturbations in directionality. Moreover, we quantify how graph-theoretic measures are affected at both the node and the network level and determine whether false positive or false negative unidirectional connections are more detrimental to the characterization of graph-theoretic measures.

## MATERIALS AND METHODS

### Connectivity Data

**Figure 1.**
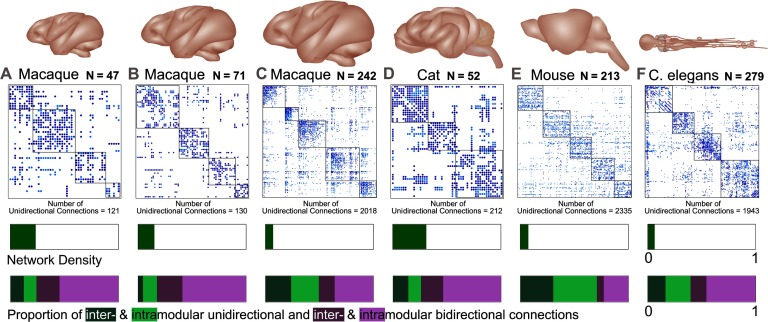
The six connectomes analyzed in this study. Brain and connectome for three different parcellations of the macaque cortex (A) nodes *N* = 47 (Honey et al., 2007), (B) *N* = 71 (Young, 1993), and (C) *N* = 242 (Harriger et al., 2012), as well as three additional species including a (D) cat (Scannell et al., 1999), (E) mouse (Dong, 2008), and (F) *C. elegans* (White et al., 1986; Varshney et al., 2011). The connectomes represent connectivity matrices with rows and columns denoting brain regions (or nodes), and the elements within the matrices denoting the presence (filled) or absence (blank) of a connection between two regions. Unidirectional connections are highlighted in light blue (with the number of unidirectional connections stated below each connectome) and the nodal regions are arranged into modular communities. The bars below each connectome display the density of each network (A = 0.234, B = 0.15, C = 0.07, D = 0.308, E = 0.073, F = 0.063) and the proportion of unidirectional and bidirectional connections. The latter is segmented to display the proportion of unidirectional connections between modules (dark green: A = 0.123, B = 0.046, C = 0.238, D = 0.142, E = 0.304, F = 0.165) and within modules (light green: A = 0.117, B = 0.129, C = 0.255, D = 0.117, E = 0.404, F = 0.232) separately, as well as the proportion of bidirectional connections between modules (dark purple: A = 0.214, B = 0.236, C = 0.147, D = 0.21, E = 0.064, F = 0.147) and within modules (light purple: A = 0.547, B = 0.59, C = 0.359, D = 0.536, E = 0.229, F = 0.457).

Following a comparative connectomics approach (van den Heuvel et al., 2016), we analyzed structural connectivity data from several species and various parcellations including three macaque connectomes, a cat and mouse connectome, and a *C. elegans* nervous system connectome (Figure 1). Each network possesses a different number of nodes, proportion of unidirectional connections, modularity, and network density (see Supplementary Table 1, Kale, Zalesky, & Gollo, 2018). Crucially, these networks include information on the directionality of connections (all networks are directed) obtained through invasive techniques that have different proportions of connection reciprocity (Garlaschelli & Loffredo, 2004). Among the meso- and macroscale connectomes, nodes represent cortical regions and the directed connections represent axons or white matter fibers linking these regions via chemical synapses. In the case of the microscale *C. elegans* connectome, nodes represent neurons, the directed connections represent chemical synapses, and the electrical synapses (or gap junctions) are bidirectional connections.

To accommodate the analysis of such a wide range of directed connectomes, the strength of connections was disregarded (for the cat and mouse connectomes) to make each network binary. This procedure allowed us to characterize all connectomes using the same methods for binary and directed networks as a first step to understand the role of directionality in structural brain networks. Other high-quality weighted connectomes can be used in future studies (Bezgin, Vakorin, van Opstal, McIntosh, & Bakker, 2012; Gămănuţ et al., 2017; Markov et al., 2012; Shih et al., 2015; Ypma & Bullmore, 2016). As recently reported, the combination of both directionality and weight can be crucial to uncover relationships between structural connectivity and univariate brain dynamics (Sethi, Zerbi, Wenderoth, Fornito, & Fulcher, 2017).

#### Macaque networks.

The first macaque network (with number of nodes *N* = 47 and connections *E* = 505, Figure 1A), used in a study by Honey, Kötter, Breakspear, and Sporns (2007), follows the parcellation scheme of Felleman and Van Essen (1991) including the visual and sensorimotor cortex, and motor cortical regions. Relevant data were collated in the CoCoMac database (Modha & Singh, 2010) following the procedures of Kötter (2004) and Stephan et al. (2001), and translated to the brain map using coordinate independent mapping (Kötter & Wanke, 2005; Stephan, Zilles, & Kötter, 2000).

The second macaque connectome (*N* = 71 and *E* = 746, Figure 1B) was derived from a whole cortex model generated by Young (1993) with regions of the hippocampus and amygdala eliminated. The parcellation was based mostly on the scheme by Felleman and Van Essen (1991), except for the fields of the superior temporal cortex (Yeterian & Pandya, 1985). Yeterian and Pandya (1985) utilized an autoradiographic technique (radioactively labeled amino acids) to establish the existence and trajectory of fibers.

The final macaque connectome (*N* = 242 and *E* = 4,090, Figure 1C) was generated by Harriger et al. (2012). This network comprises anatomical data from over 400 tract tracing studies collated in the CoCoMac database (Modha & Singh, 2010) following the procedures of Kötter (2004) and Stephan et al. (2001), focusing on the right hemisphere with all subcortical regions removed as well as regions without at least one incoming and one outgoing connection.

The data collated for the CoCoMac database used a range of tracer substances (with anterograde, retrograde, or bidirectional transport properties) and methods (as discussed in Stephan et al., 2001). Each contributing study must discern a source and target for the connection. If the reciprocal direction had not been tested for, the connection was assumed to be unidirectional. Some connections have been confirmed to be unidirectional, for example, the connection from V2 to FST; see Boussaoud, Ungerleider, and Desimone (1990). Regarding macaque connectomes, Felleman and Van Essen (1991) have also suggested that the reciprocity of connections may vary between individuals.

#### Cat network.

The cat matrix is a connectome reconstructed by Scannell et al. (1999) and curated from a database of thalamo-cortico-cortical connections from a large number of published studies in the adult cat. The parcellation was based on a previous scheme by Reinoso-Suarez (1984) and adapted by Scannell, Blakemore, and Young (1995). Areas ALG, SSF, SVA, DP, Amyg, and 5m were discarded (and some regions grouped) to create a weighted network (*N* = 52 and *E* = 818, Figure 1D). This connectome was generated from the available data across numerous studies. It is noted that each study used a different type of anterograde and/or retrograde tracer, methodology, and parcellations. Some connections lacked data on the existence of a reciprocal direction between brain regions (these were left as unidirectional), and all connections between the cortex and thalamus were assumed to be reciprocal.

#### Mouse network.

We obtained the mouse connectome (*N* = 213 and *E* = 2,105, Figure 1E) from the Allen Mouse Brain Connectivity Atlas generated by Dong (2008). The major advantage of this connectome is that the connectivity data, obtained at a cellular level (axons and synaptic terminals), are generated for the whole mouse brain. Therefore, all 469 individual experiments use the same anterograde tracer and consistent techniques. Each brain is applied to a 3D template, which itself is averaged across 1,231 brain specimens, and the regions are matched against the Allen reference atlas (Dong, 2008). We thresholded this dense and weighted network using the disparity filter (Serrano, Boguná, & Vespignani, 2009), maintaining only connections with a *p* value smaller than 0.05. Thresholding was performed such that the resulting network was binary.

#### *C. elegans* network.

The *C. elegans* nervous system matrix (*N* = 279 and *E* = 1,943, Figure 1F) was collated by Varshney, Chen, Paniagua, Hall, and Chklovskii (2011), and includes data mapped by White, Southgate, Thomson, and Brenner (1986) using electron microscopy, in addition to various other sources (Durbin, 1987; Hall & Russell, 1991; White, Southgate, Thomson, & Brenner, 1976). This microscale connectome is composed of a directed chemical synapse network and an undirected gap junction network. Although gap junctions may possess directionality, this has not yet been demonstrated in *C. elegans*. For the purpose of analysis, the connections from the gap junction network were treated as bidirectional connections.

### Perturbed Networks

**Figure 2.**
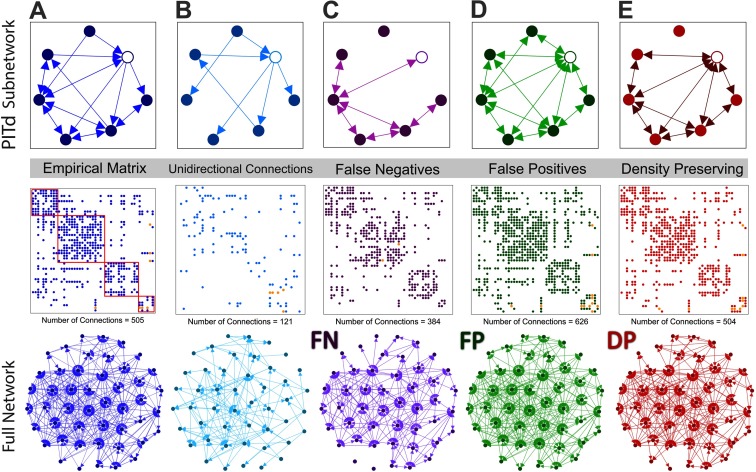
Structural connectome for the macaque *N* = 47 cortex and perturbed undirected variants, with an exemplar subnetwork. Subnetwork (top) encompassing the PITd region (white node) and neighboring nodes, the adjacency matrix (middle), and the entire network (bottom) for (A) macaque empirical connectome with the community modules outlined in red; (B) unidirectional connections of the connectome; (C) connectome with unidirectional connections removed (false negative network); (D) connectome with reciprocal connections added to unidirectional connections (false positive network); (E) connectome with one randomly selected reciprocal connection added to a unidirectional connection for each randomly selected unidirectional connection removed (density-preserving network). In each connectome, the connections linking PITd (dorsal posterior inferotemporal) to the rest of the network are colored orange.

To investigate the effects of directionality on the characteristics of the brain, each empirical connectome was altered by progressively removing connection directionality information, generating a spectrum of perturbed networks. This spectrum comprised the empirical connectome at one end, and a fully undirected representation of the connectome at the opposite end. For this purpose, the empirical networks were considered to be approximately the ground-truth connectomes for a given parcellation. Figure 2 illustrates the three different approaches used to generate perturbed networks for the macaque (*N* = 47) connectome. The empirical connectome is shown in Figure 2A, and the unidirectional connections of this network are shown in Figure 2B. Perturbed networks (Figures 2C–E) were generated by altering the directionality or presence of the unidirectional connections. In this example, we only show the extreme case in which all information about connection directionality is removed, yielding a fully undirected perturbed network.

For further analyses we present three schemes that were developed to progressively eliminate connection directionality information from the empirical connectomes, yielding perturbed networks that increasingly resembled undirected networks.

#### False negative perturbed networks.

The first perturbed network was generated by removing a fixed number of randomly chosen unidirectional connections, leading to a connectome with false negative unidirectional connections (FN network, Figure 2C). The perturbed network was undirected in the extreme case when all unidirectional connections were removed. This perturbation assumes that unidirectional connections are weaker in strength (weight) relative to their bidirectional counterparts, and thus unidirectional connections are most vulnerable to elimination with weight-based thresholding procedures (Rubinov & Sporns, 2010). Such thresholding is commonly used to eliminate weak connections obtained with tractography, which are often attributed to noise or error (Maier-Hein et al., 2017). As an example, the majority of the weighted mouse connectome is composed of unidirectional connections (57%), and they are also weaker than the bidirectional connections. The mean of the strength of unidirectional connections is 0.066, whereas the mean strength of bidirectional connections is 0.165, which is significantly weaker (*P* < 10^−45^, Welch’s *t* test).

#### False positive perturbed networks.

If the weight of a unidirectional connection exceeds the weight-based threshold, the connection will be represented in the perturbed network as an undirected connection (i.e., a unidirectional connection from node *u* to *v* becomes an undirected connection between nodes *u* and *v*). In this case, the undirected connection is treated as a bidirectional connection, and thus construed as a false positive. To model this case, we generated perturbed networks by adding reciprocal connections to a fixed number of randomly chosen existing unidirectional connections, leading to a perturbed network with false positive reciprocal connections (FP network, Figure 2D). In the extreme case when all reciprocal connections were added, the perturbed network effectively became an undirected network.

#### Density-preserving perturbed networks.

Finally, to preserve basic properties of the empirical connectome, an additional perturbed connectome termed the density-preserving network was generated (DP network, Figure 2E). In this perturbed connectome, for each reciprocal connection added to a unidirectional connection, another unidirectional connection is removed (at randomly selected locations). The DP network has an equal number of false negative and positive connections and also preserves the mean degree of the empirical connectome, but not the degree of each node.

To generate undirected perturbed networks, we progressively applied one of the above three schemes to randomly chosen unidirectional connections in the empirical connectomes until a desired proportion of connections were changed. We generated perturbed networks in which 5%, 10%, 20%, and 100% of directed connections were altered (eliminated or the reciprocal connection added). This process was repeated for multiple trials to generate an ensemble of perturbed networks. Ensemble averages for all graph-theoretic measures were then computed. Each perturbed network was associated with a rewiring scheme (FN, FP, and DP) and a proportion of changed connections. Supplementary Table 2 (Kale et al., 2018) provides the details of the proportion of unidirectional connections altered in the perturbed networks and other relevant parameters used for each analysis.

The perturbed networks can comprise isolated nodes that are not connected to any other nodes (see Supplementary Figure 1, Kale et al., 2018). Isolated nodes are more likely to occur in the FN perturbed networks, potentially having a greater impact on graph-theoretic measures as more connections are changed. Therefore, in cases where only a subset of unidirectional connections are modified (<100%), the trials that cause nodes to become disconnected are rejected.

### Network Measures

Connectome analyses were performed using a range of common graph-theoretic network measures (da Fontoura Costa, Rodrigues, Travieso, & Villas Boas, 2007). These measures enable the quantitative comparison of connectomes across species and neuroimaging techniques while remaining computationally inexpensive (Rubinov & Sporns, 2010). Furthermore, the graphical properties of cortical systems have previously been associated with functional connectivity and evolutionary adaptations in behavior and cognition (Bullmore & Sporns, 2012; van den Heuvel et al., 2016). For each empirical connectome and associated perturbed network, we computed several graph-theoretic measures (see Supplementary Table 3, Kale et al., 2018), using the Brain Connectivity Toolbox (Rubinov & Sporns, 2010). Graph-theoretic measures for directed networks were used in all cases where applicable.

#### Measures of centrality.

The *degree* of each node was calculated as the sum of the in- and out-degree, or the sum of all directed connections connecting that node to the rest of the network (Rubinov & Sporns, 2010). Network centrality identifies nodes that act as important points of information flow between regions. We used a *betweenness centrality* measure, defined as the fraction of all the shortest paths between regions that pass through a particular node (Freeman, 1978). The *participation index* or *coefficient* describes the proportion of intra- and intermodular connections linking each node (Guimera & Amaral, 2005a). As shown in Supplementary Table 3 (Kale et al., 2018), we used the out-participation index with the Louvain algorithm (Blondel, Guillaume, Lambiotte, & Lefebvre, 2008) to define network modules (Rubinov & Sporns, 2010). Further details about module delineation are provided below.

#### Measures of functional segregation.

We calculated the *clustering coefficient*, a measure describing the proportion of a node’s neighbors that are connected to each other (Fagiolo, 2007). In undirected networks it is calculated as the probability that two connections (linking three nodes) will be closed by a third connection to form a triangle. In directed networks, however, a set of three nodes can generate up to eight different triangles. The function utilized in this study, clusteringcoef_bd (Rubinov & Sporns, 2010), takes this into account.

#### Measures of functional integration.

A path is defined as a sequence of nodes and connections that represent potential routes of information flow between two brain regions. In a directed network, connections comprising a path must be arranged such that the head of one connection always precedes the tail of the subsequent connection. The *characteristic path length* for each network was calculated as the average shortest distance between all pairs of nodes (Watts & Strogatz, 1998). We also calculated the *global efficiency* of each network as the average nodal efficiency, which is the reciprocal of the harmonic mean of the shortest path length between all pairs of nodes (Latora & Marchiori, 2001).

#### Small-worldness.

Lastly, we measured the small-world characteristics of each network (Watts & Strogatz, 1998). For each node and for the network (see Supplementary Table 3, Kale et al., 2018), the *small-world index* was classified as the clustering coefficient divided by the characteristic path length of the network, with a comparison to a directed random network, makerandCIJ_dir (Rubinov & Sporns, 2010), unless otherwise stated (Humphries & Gurney, 2008). This index combines local and global topological properties and has been linked to network efficiency (Bassett & Bullmore, 2006).

#### Community detection and modularity.

We generated consensus matrices to describe the community structure of each empirical connectome (Lancichinetti & Fortunato, 2012). Specifically, 100 runs of the Louvain modularity algorithm (Blondel et al., 2008) were performed to generate a set of modular decompositions for each empirical connectome. The different runs did not necessarily yield identical decompositions because of degeneracy of the solution space and the stochastic nature of the algorithm. A consensus modularity matrix was determined for the 100 decompositions such that each element in the consensus matrix stored the proportion of runs for which a particular pair of nodes comprised the same module. The consensus modularity matrix was then thresholded (retaining values >0.4), and 100 runs of the Louvain algorithm were performed on the thresholded consensus matrix. This process was iterated until the consensus matrix converged and did not change between successive iterations. The macaque *N* = 47 network required a greater number of iterations before a consistent community structure could be achieved (macaque *N* = 47: 408, macaque *N* = 71: 2, macaque *N* = 242: 5, cat: 4, mouse: 36, *C. elegans*: 2).

For the perturbed networks with all unidirectional connections altered, a single consensus matrix and consistent modularity was obtained for the FN and FP networks. For the rank correlation-coefficient analyses, the modularity for each perturbed network remained the same as that assigned to the associated empirical connectome. These perturbed networks only had a small percentage of unidirectional connections altered (5%). With these measures we intended to isolate the effect of directionality on the ranking of nodes by each graph-theoretic measure, and, therefore, used the empirical consensus modularity for the (participation index) calculations on each type of perturbed network.

For DP networks with 100% of connections altered, a consensus matrix was obtained for each trial (see Supplementary Table 2 for more details; Kale et al., 2018). For other perturbed networks where 5%, 10%, and 20% of unidirectional connections are altered, consensus modularity matrices were obtained for each run (50 runs; see Supplementary Table 2, Kale et al., 2018) and for each type of network (FN, FP, and DP).

### Classification of Highly Connected Regions

Core nodes were determined using the core-periphery algorithm, function core_periphery_dir from the Brain Connectivity Toolbox (Rubinov & Sporns, 2010), with gamma = 1, which subdivides all nodes in the network into either core or periphery groups of similar size. Hubs were defined as regions with a degree at least one standard deviation above the mean (Sporns et al., 2007), and super hubs were classified as those with a degree of at least 1.5 standard deviations above the mean (see Figure 4A for an example). Super hubs were defined to evaluate the robustness of hub nodes to the progressive removal of connection directionality. More specifically, we aimed to assess whether super hubs would be demoted to hubs or nonhub nodes as directionality information was lost.

We tested the resilience of the classification of nodes belonging to the core of the network, or the set of hubs and super hubs. For each perturbed network, the accuracy of the classification of nodes into each of these three groups (core, hubs, and super hubs) was compared with the empirical connectomes. For each group, the accuracy, or matching index, *A* was computed taking into account the number of nodes with common classification and the number of mismatched nodes that had a different classification between the empirical and the perturbed networks. More precisely, *A* was given by the simple matching index:

$$A=\frac{C}{C+\left(N_{e}-C\right)+(N_{b}-C)},$$ (1)

where *C* was the number of overlapping nodes within the same group between the empirical and perturbed networks; *N*_*e*_ was the number of nodes within this group for the empirical connectome; and *N*_*b*_ was the number of nodes within this group for the perturbed network. This measure of accuracy attained a minimum of 0 when there was no overlap between the connectomes and a maximum of 1 for a perfect overlap.

The participation index can be used to classify nodes, and has been applied to hubs (Guimera & Amaral, 2005b). Hubs with large participation index connect areas from different modules. Supplementary Table 4 (Kale et al., 2018) lists the regions classified as hubs for each empirical network, as either connector (with a participation index *Y* > 0.35) or provincial (*Y* ≤ 0.35) hubs. Consistent with other studies (Sporns et al., 2007), node degree (as the sum of the in- and out-degree) was used to define the set of hubs based on their topological role within the network.

### Quantifying Changes in Network Measures

To investigate changes in node-specific features between the empirical connectomes and corresponding perturbed networks, we developed a measure to quantify the change in the ranking of nodes. Nodes can be ranked with any of a number of graph-theoretic measures. The rank-shift index (RSI) represents the sum of the absolute value of the difference between the ranking of the empirical (*E*) and perturbed (*B*) matrices for each node, divided by the maximum possible difference (*D*) in which the ranks of the network are reversed:

$$\text{RSI}=\sum_{i=1}^{\text{N}}\frac{\left|{\text{E}}_{\text{i}}-{\text{B}}_{\text{i}}\right|}{\text{D}}.$$ (2)

An RSI of 0 indicates no change, and an index of 1 indicates a complete inversion in the rank order (see Figure 5). Node-level changes were also measured by the Spearman rank correlation (Spearman, 1904) and Kendall coefficient (Kendall, 1938).

## RESULTS

To understand the effects of neglecting connection directionality on the structural properties of connectomes, we compared several directed brain networks across multiple species, including three macaque connectomes (with different parcellation schemes), a cat, a mouse, and a *C. elegans* connectome. The characteristics of each of these networks were analyzed using a range of network measures: degree, betweenness centrality, clustering coefficient, characteristic path length, global efficiency, participation index, and small-world index.

**Figure 3.**
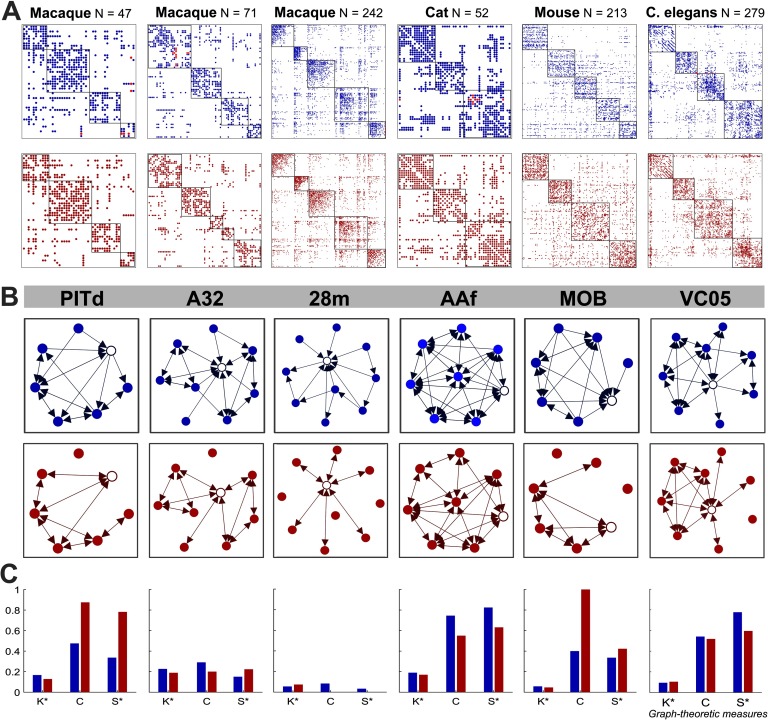
Graph-theoretic measures for a specific region of interest from each empirical and density-preserving connectome. (A) Empirical (blue) and density-preserving (red, an illustrative single trial with 100% of unidirectional connections altered) connectomes. Nodal regions are arranged into modular communities and the connections connecting the region of interest to the rest of the network in the empirical connectome are colored red. (B) Labels for each region of interest (top), and subnetworks of the local neighborhood around each region of interest (white node). (C) Graph-theoretic measures at the selected brain region for the empirical and density-preserving networks. Graph-theoretic measures are as follows: K = degree, C = clustering coefficient, and S = small-world index ($S_{i}^{\to }$). *Normalized by the maximum value of that measure across all nodes in their respective network. PITd: dorsal posterior inferotemporal, A32: anterior cingulate area 32, 28m: medial entorhinal cortex, AAF: anterior auditory field, MOB: main olfactory bulb, VC05: ventral cord neuron 5.

We altered unidirectional connections according to one of three schemes (see Methods) to progressively eliminate information about connection directionality. We then quantified the inaccuracies in graph-theoretic measures admitted through this loss of directionality information. We begin with the density-preserving (DP) scheme and consider the extreme case in which all unidirectional connections are eliminated, resulting in an undirected network. In particular, we compare the network characteristics of selected regions of interest (ROIs) across the empirical connectomes and single-trial DP counterparts (Figure 3). These ROIs (shown as the red matrix entries in Figure 3A) occupy peripheral locations in the network topology and have low degree, and the subnetwork of the local neighborhood surrounding each ROI can be clearly represented (Figure 3B). From the empirical to the DP subnetworks, unidirectional connections are eliminated and made bidirectional, resulting in changes to graph-theoretic measures characterizing these regions. Figure 3C illustrates the relative graph-theoretic metrics at these exemplar regions for the empirical and DP subnetworks. Although the mean degree of the DP network is preserved, at the node level, the degree may increase or decrease depending on whether the unidirectional connections surrounding the node of interest received more false positive or false negative alterations. Likewise, clustering and small-worldness also exhibit trial-dependent changes based on how the neighbors of these exemplar regions and the whole network topology are affected.

### Highly Connected Regions

Connectivity across brain regions and connections is heterogeneously distributed. Hub nodes are identified as the most connected neural regions, and have enhanced importance in information integration for cognitive functions (van den Heuvel & Sporns, 2013). Hub nodes can be further classified based on their participation index as either provincial or connector hubs depending on their level of intra- versus intermodule connectivity (Guimera & Amaral, 2005b; Sporns et al., 2007). Provincial hubs with a high intramodule degree and low participation index, are thought to facilitate modular segregation. Conversely, connector hubs, with a higher participation index, are thought to assist with intermodular integration (Rubinov & Sporns, 2010). When hub regions are more densely connected among themselves than to other nodes they form a “rich club,” consisting of a central but costly backbone of pathways that serve an important role in global brain communication (Aerts, Fias, Caeyenberghs, & Marinazzo, 2016; Colizza, Flammini, Serrano, & Vespignani, 2006; van den Heuvel et al., 2012). Hence, alterations to directionality at hub nodes influence the network activity observed in functional connectivity. But how is the identification and characteristics of these highly significant hub regions affected when directionality is modified?

**Figure 4.**
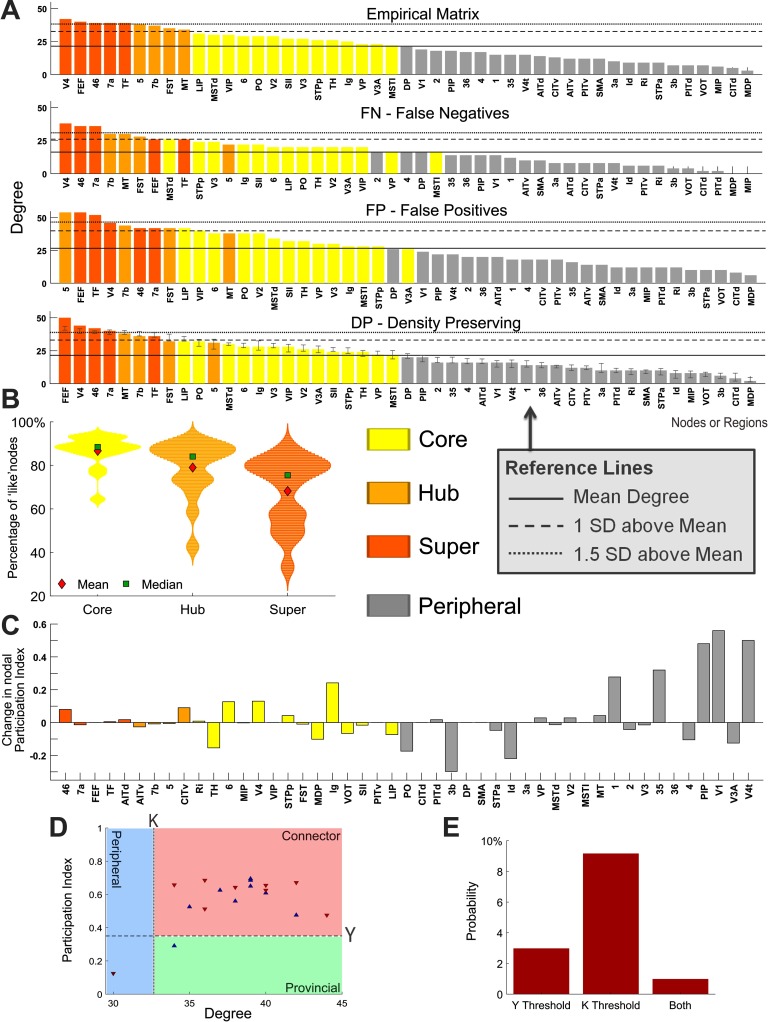
Identification of hubs, changes in graph-theoretic measures at the node level, and provincial/connecter hub classification. (A) Cortical areas of the macaque *N* = 47 connectome sorted by degree for the empirical and each perturbed network. Hubs are defined as nodes that have a total degree (in-degree plus out-degree) at least 1 standard deviation above the mean, and super hubs are defined as nodes that have a degree at least 1.5 standard deviations above the mean. The density-preserving results are from an illustrative single trial and show the standard deviation in degree for each node (over 1,000 trials). (B) Percentage of core, hub, and super-hub nodes across the perturbed networks of all six connectomes that retain correct classification according to their empirical connectome (as the mean over 1,000 trials). (C) Change in the participation index of each brain region from the empirical macaque *N* = 47 connectome to an illustrative case of the density-preserving network. (D) Identification and classification of hub nodes for the empirical (blue) macaque *N* = 47 connectome and an illustrative case of the density-preserving (red) network. The dotted line represents the hub definition based on the degree, and the dashed line represents the subclassification of hubs as either connector (*Y* > 0.35) or provincial (*Y* ≤ 0.35), based on the participation index. (E) Mean probability (across all connectomes over 1,000 trials) that hub nodes will cross over either, or both, of the threshold lines following density-preserving alterations in directionality, resulting in a classification that is inconsistent with the empirical connectomes. (A–E) Each perturbed network has 100% of unidirectional connections altered. Hub nodes are defined in the empirical network and retain the same definition in the perturbed networks.

Inaccuracies may be introduced to node-specific graph-theoretic measures as connection directionality information is lost. By comparing the empirical connectomes to corresponding perturbed networks with all unidirectional connections eliminated according to the DP scheme, we see that peripheral, core, and hub nodes are all impacted (Figure 4). Even the degree, a fundamental network characteristic, is affected in these perturbed networks, as shown in Figure 4A for each cortical area in the macaque *N* = 47 connectome. In particular, the degree of some hub and super-hub nodes falls below the threshold used for their classification in the empirical connectome. This implies that hub nodes identified based on degree can be inaccurate when directionality within the network is neglected or unknown. To further investigate this, we redefined core, hub, and super-hub nodes for each perturbed network, and calculated their accuracy according to the empirical connectome. Figure 4B shows the percentage of nodes that retain the same classification for core, hub, and super-hub nodes across all perturbed networks. We find that the estimation of core nodes from the perturbed networks was the most accurate compared with the empirical connectomes (mean = 86.7%). However, the estimation of hubs and super hubs is less precise (mean = 79% and 68.2%, respectively). The accuracy of nodes belonging to core, hub, and super-hub was tested with paired sample *t* tests and found to be significantly different. Core (including results from all connectomes and each type of perturbed network) versus hubs *P* = 0.0027, core versus super hubs *P* = 0.00001, and hubs versus super hubs *P* = 0.003. In Supplementary Figure 2 (Kale et al., 2018) these results are shown for each type of perturbed network and connectome separately.

A recent study in the mouse brain (Sethi et al., 2017) showed a strong correlation between the in-degree characteristics of a brain region and its resting-state functional MRI dynamics. We therefore sought to investigate in- and out-degree separately. Supplementary Figures 3A and 3B (Kale et al., 2018) display the in- and out-degree of all cortical regions in the macaque *N* = 47 empirical connectome and perturbed networks. In this case, the delineation of hubs and super-hub nodes depends on the directed degree, and therefore a different set are identified in Figures 2A and 2B (Kale et al., 2018). However, because of the methodology for generating the perturbed networks, the resulting in- and out-degree of each node becomes equal. This is because (when 100% of unidirectional connections are altered) the only remaining connections in each case (FN, FP, or DP) are represented as bidirectional, and therefore each region has the same number of incoming connections as it has outgoing connections. Previous studies in the cat connectome have found that high in-degree nodes also show (on average) a high out-degree as well. In this connectome, 66% of rich-club nodes (defined by the summed degree) had a higher in-degree than out-degree (de Reus & van den Heuvel, 2013). A comparison across the connectomes analyzed in this study (Supplementary Figure 3C, Kale et al., 2018) showed that four out of six sets of hub regions had a higher mean in-degree than out-degree. The mouse connectome, however, was an interesting case for which all hub regions had a much larger out-degree.

Next, we investigate the classification of hubs based on the participation index. In comparison to peripheral regions, the participation index of hub nodes is more resilient as illustrated in Figure 4C as the change for each region from the empirical macaque *N* = 47 connectome to a (typical) DP example network. Because peripheral nodes have a low degree, the alterations in directionality may affect a larger proportion of these connections. Therefore, peripheral regions often show greater change in the participation index than both core and hub nodes. As illustrated in Supplementary Figure 4 (Kale et al., 2018), this also occurs for other graph-theoretic measures.

The relationship between participation index and degree for the set of hub nodes (defined in the empirical connectome) are displayed in Figure 4D for the empirical macaque *N* = 47 connectome and an illustrative DP network. Directionality alterations to the network cause changes in these measures, both of which were used to define and classify the set of hubs in the empirical connectome. As such, some of these regions in the DP network exceed the degree and participation index thresholds (degree *K* = 1 *SD* above the mean and *Y* = 0.35), resulting in misclassifications according to the empirical network. Across all connectomes, hub nodes are more likely to lose their classification based on degree, indicating that the definition of hubs based on the degree is on average 3.5 times more vulnerable to changes in directionality in comparison to the misclassification of hubs based on the participation index (Figure 4E and Supplementary Figure 5, Kale et al., 2018). Supplementary Figure 6 (Kale et al., 2018) displays the number of core, hub, and super hubs across the connectomes (A: mean, B: individually), as defined in the empirical and each perturbed network.

### Quantifying the Errors in Node Rank When Directionality Is Lost

**Figure 5.**
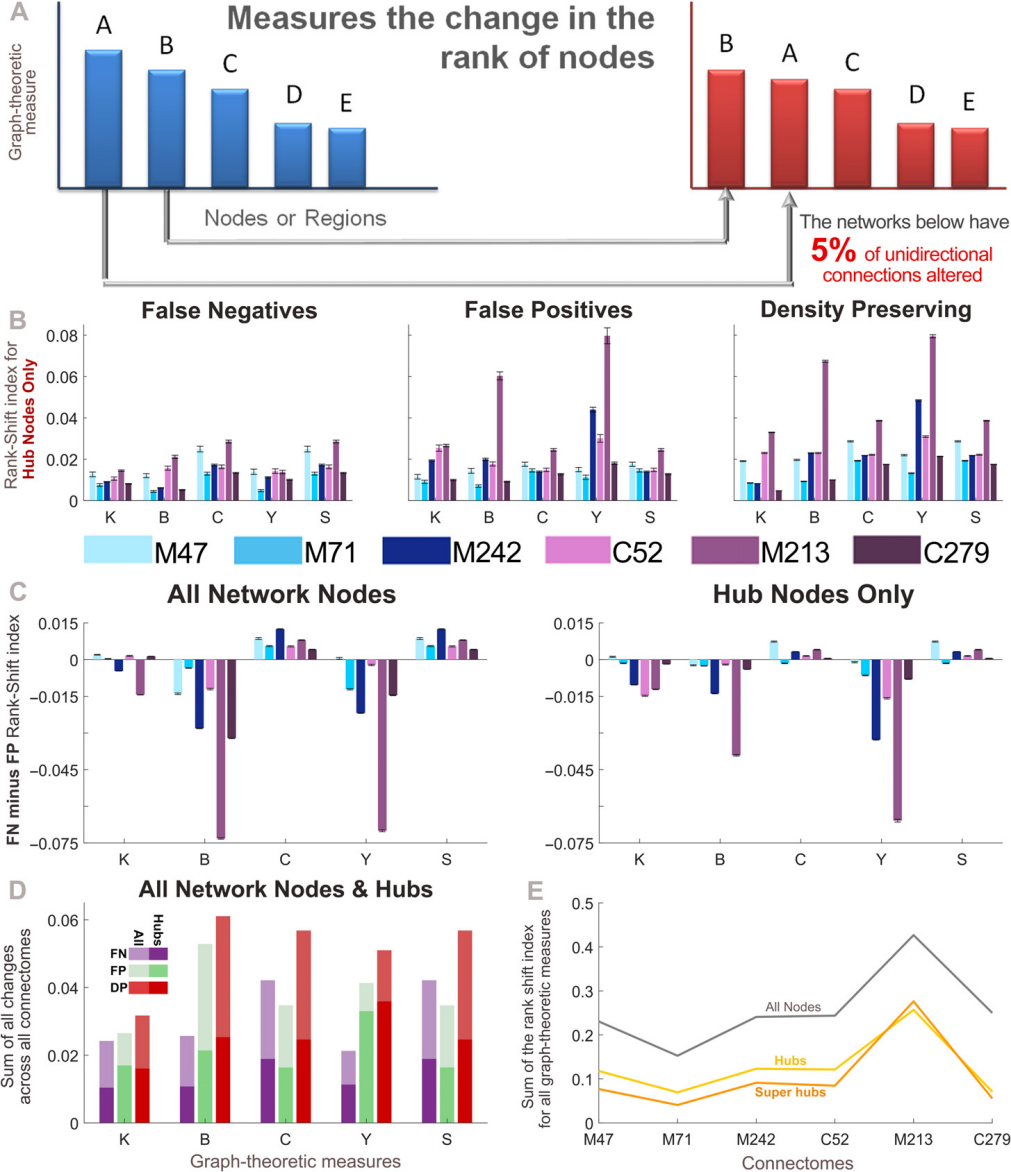
Nodal changes measured by the Rank-Shift Index. (A) The rank-shift index quantifies the change in the rank of nodes from the empirical connectome to the perturbed network when they are ordered by a particular graph-theoretic measure. More specifically, it calculates the sum of the difference between graph-theoretic values for each node in the empirical and perturbed matrices, divided by the maximum potential difference that could exist between these two networks (where a value of 0 indicates no change, and a value of 1 indicates the maximum change). See Methods for further explanation. (B) Rank-shift index of hub nodes across all perturbed networks, for each graph-theoretic measure. (C) Difference in the rank-shift index between the false negative and false positive networks for all nodes (left), and hub nodes (right). A positive value indicates that the false negative connections cause greater changes in the ranking of nodes, whereas a negative value indicates the same for false positive connections. (D) Rank-shift index for each graph-theoretic measure summed across all connectomes. (E) Rank-shift index values summed across all graph-theoretic measures for each density-preserving connectome. (B–E) Results correspond to the mean over 50 trials for which 5% of randomly selected unidirectional connections are modified in each perturbed network (error bars show the standard error of the mean). Graph-theoretic measures are as follows: K = degree, B = betweenness centrality, C = clustering coefficient, Y = participation index, and S = small-world index ($S_{i}^{\to }$). M47: the macaque connectome with 47 nodes, M71: macaque *N* = 71, M242: macaque *N* = 242, C52: cat, M213: mouse, C279: *C. elegans*.

All the results presented thus far have pertained to perturbed networks in which all unidirectional connections are altered, yielding perturbed networks that are effectively undirected. Next, we investigate the impact of losing only a small proportion of connection directionality information. To this end, we generate perturbed networks in which the proportion of unidirectional connections altered is 5%. Changes in node-specific network measures were quantified using the rank-shift index (RSI, see Methods). This measure calculates the change in the ranking of nodes by a specific graph-theoretic measure from the empirical to the perturbed networks (see Figure 5A). We first focus on the set of hub nodes for each connectome, finding that differences in the RSI can be seen across perturbed networks and graph-theoretic measures (Figure 5B; super-hub results were similar). Figure 5C directly compares the effects of the FN and FP connections (perturbations) on the graph-theoretic measures, first across all nodes in the network, and then for the set of hub nodes. It can be seen that the FP connections consistently have a greater effect on the betweenness centrality and participation index, whereas the clustering coefficient and small-worldness are more affected by the FN connections. For hub nodes, the RSI shows that the degree is also more affected by FP connections.

The RSI calculation is similar to the Spearman rank correlation coefficient (Spearman, 1904) and Kendall rank coefficient (Kendall, 1938) at the network level. Supplementary Figure 7 (Kale et al., 2018) pertains to analyses repeated with these similar, yet alternative, measures and should be compared with Figures 5B and 5C. Regardless of the measure used, the overall trends in the data between Figures 5B and 5C and Supplementary Figure 7 are consistent.

Directly comparing each of the methods for altering directionality (Figure 5D), we find that the DP networks showed the greatest RSI across almost all measures. Across connectomes the summed RSI for all graph-theoretic measures were quite similar (Figure 5E). In particular, the mouse connectome, which has the largest proportion of unidirectional connections (see Figure 1 and Supplementary Table 1), showed larger differences for the same percentage of altered connections.

### Quantifying the Importance of Directed Connections in the Whole Network

**Figure 6.**
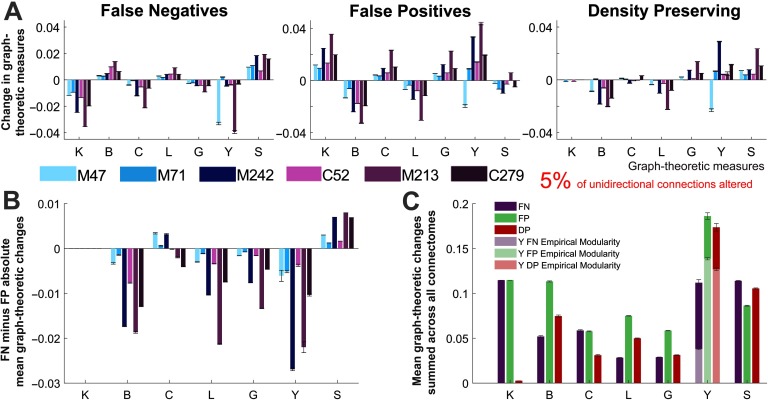
Relative changes in mean graph-theoretic measures for perturbed networks. (A) Changes in mean graph-theoretic measures across all connectomes and each type of perturbed network. (B) Difference between the changes in mean graph-theoretic measures for the false negative and false positive networks. (C) Mean changes in graph-theoretic measures for each of the perturbed networks, summed across all connectomes. Two separate modularity inputs are used the participation index calculations for the perturbed networks: the consensus modularity of the empirical networks (light colors) and the new modularity assignments for each generated perturbed network (dark colors). (A–C) All results correspond to perturbed networks with 5% of randomly selected unidirectional connections modified. The results represent the mean of these networks over 50 trials, and describe the change in the mean graph-theoretic measure (from the empirical to perturbed network) normalized by the mean of the empirical network (error bars show the standard error of the mean). Graph-theoretic measures are as follows: K = degree, B = betweenness centrality, C = clustering coefficient, L = characteristic path length, G = global efficiency, Y = participation index, and S = small-world index (*S*^→^, changes in this measure are presented as the mean over 1,000 trials). M47: the macaque connectome with 47 nodes, M71: macaque *N* = 71, M242: macaque *N* = 242, C52: cat, M213: mouse, C279: *C. elegans*.

We next considered the mean changes in graph-theoretic measures in the whole network caused by the loss of directionality. We focus our analysis on perturbed networks with alterations to a small percentage of the unidirectional connections (5%; see Figure 6). In the initial two perturbed connectomes, false negative and false positive alterations have opposite effects on network measures (Figure 6A). The changes in betweenness (B), characteristic path length (L), and global efficiency (G) are directly dependent on the degree (K), as these connections facilitate a shorter route between nodes. The effects pertaining to clustering (C), participation index (Y), and small-world index (S) are more complex because they depend on whether the changes increase or decrease the interneighbor or the intermodular connectivity. Aside from the mean degree (which is preserved in the DP networks), the effects on graph-theoretic measures were mostly similar across the FP and DP perturbed networks. To better understand the role of unidirectional connections, we next compare how false positive and false negative modifications affect the mean graph-theoretic measures of networks (Figure 6B). When it is not possible to distinguish the directionality of the connections, is it better to assume that they are bidirectional or to disregard unidirectional connections?

In the case where a subset of connections is altered, for most graph-theoretic measures the false positive unidirectional connections were more detrimental. It can be seen in Supplementary Figure 8 (Kale et al., 2018) that this trend remains robust as the proportion of unidirectional connections is increased (to 10% and 20%). However, the error present in each graph-theoretic measure is predictably increased. With the exception of the small-worldness and degree, the FP perturbed networks consistently show the greatest changes in the mean graph-theoretic measures (Figure 6C and Supplementary Figures 8C and 8F, Kale et al., 2018). The participation index is the only measure directly affected by the modularity of the networks.

The changes in mean graph-theoretic measures are emphasized across connectomes in Supplementary Figure 9 (Kale et al., 2018). In the FN and FP networks, the changes for each graph-theoretic measure depend on the degree and proportion of unidirectional connections. Once again, the degree is correlated with the global efficiency and inversely correlated with the characteristic path length and betweenness. Moreover, the clustering coefficient is also correlated with the changes in degree, but this is caused by the elimination of triangles from false negatives and addition of triangles from false positives.

## DISCUSSION

Over 10 years ago, Sporns et al. (2005) proposed an influential coordinated research strategy to map the human connectome, which motivated and guided many researchers. A lot of progress has been made towards this goal with the development of diffusion-weighted imaging and tractography methods, enabling the reconstruction of several descriptions of the human connectome (Assaf & Basser, 2005; Goulas et al., 2014). However, much more research is needed to achieve an accurate, reliable, and standardized representation of connectivity in the human brain. It must also be acknowledged that the methods of collation and reconstruction for these large datasets, including diffusion imaging and tract tracing, can give rise to errors and inconsistencies in the data, as discussed elsewhere (Calabrese et al., 2015; Donahue et al., 2016; Gămănuţ et al., 2017). Beyond this, several parcellation schemes have been proposed for the human connectome (Cloutman & Ralph, 2012; de Reus & van den Heuvel, 2013; Glasser et al., 2016; Honnorat et al., 2015), which can each have different effects on the characterization of the network (Zalesky et al., 2010). Furthermore, the inability to resolve connection directionality noninvasively, which was originally classified as a crucial task (Sporns et al., 2005), has remained surprisingly overlooked. Without improvements in neuroimaging techniques, directionality can only be indirectly estimated for the human connectome, for example, investigating effective connectivity (Friston, 2011; Stephan, Tittgemeyer, Knösche, Moran, & Friston, 2009). With current macroscale connectome mapping techniques, connection directionality cannot be explicitly resolved.

Here, we quantified the impact of disregarding directionality in connectome analysis. Specifically, we estimated the inaccuracies in brain networks quantified by graph-theoretic measures following modifications to the unidirectional connections in connectomes of different species and parcellations.

Our analyses indicate that several network measures are susceptible to error when directionality is lost. Graph-theoretic measures are affected at both the individual-node and the network level, as is the definition of hubs. Across all networks analyzed, those with a larger proportion of unidirectional connections were more extensively affected by the loss of connection directionality. This proportion is closely related to the parcellation, as finer parcellations tend to have a larger proportion of unidirectional connections. We have also compared three different schemes to generate undirected networks, which showed that the addition of reciprocal connections to a subset of existing connections (false positives) is more detrimental to graph-theoretic measures than the removal of unidirectional connections (false negatives).

### Error in the Classification of Hub Nodes

Heterogeneity in cortical regions plays an important role in structural brain networks: Highly connected hub regions support integration of functionally and structurally segregated brain regions (Mišić et al., 2015; van den Heuvel et al., 2016; van den Heuvel et al., 2012). At these regions, neuronal dendrites have larger spine density (Scholtens, Schmidt, de Reus, & van den Heuvel, 2014; van den Heuvel & Sporns, 2013) and increased transcription of metabolic genes (Fulcher & Fornito, 2016). Moreover, hub nodes have high wiring cost and demand for metabolic resources, meaning their connections are more likely to become structurally damaged and symptomatic in a wide range of neuropsychiatric disorders (Crossley et al., 2014; Fornito et al., 2015; Fulcher & Fornito, 2016). For example, the increased vulnerability of hubs in Alzheimer’s disease could be explained by excessive neuronal activity at these regions (de Haan, Mott, van Straaten, Scheltens, & Stam, 2012; Kitsak et al., 2010; Raj, Kuceyeski, & Weiner, 2012). Hence, the correct identification and classification of hub regions is crucial to understanding the effects of their normal functioning (van den Heuvel & Sporns, 2013) and dysfunction (Fornito et al., 2015) within the brain network.

Our results indicate that a proportion of hubs and super-hub nodes of the human connectome are vulnerable to misclassification because the directionality of connections is not available. In particular, the classification of super-hub nodes was found to have a significant lower accuracy than hub nodes. As a caveat, we need to be aware that this measure is sensitive to noise because the number of super-hub nodes in some of the connectomes is limited.

Hubs were also classified as either connector or provincial based on their level of intramodule versus intermodule connectivity (Guimera & Amaral, 2005b; van den Heuvel & Sporns, 2011). Previous studies have found that targeted attacks on connector hubs have a widespread effect on network dynamics because of their role in functional integration, whereas attacks on provincial hubs produce a more localized effect within communities (Honey & Sporns, 2008). It has been hypothesized that such localized damage would cause specific clinical deficits, whereas damage to connector hubs would cause complex, distributed dysfunction throughout the network (Fornito et al., 2015). We found that alterations to unidirectional connections lead to multiple errors in the classification of hub regions. Hubs were more likely to be defined incorrectly based on degree (losing their classification) rather than the participation index (changing classification between connector and provincial).

### Effect of False Positive and False Negative Connections

Diffusion-weighted and diffusion tensor imaging allow detailed reconstructions of the structural human brain network (Iturria-Medina, Sotero, Canales-Rodríguez, Alemán-Gómez, & Melie-García, 2008; Van Essen et al., 2013). Depending on the data and specific tractography algorithms used, crossing fiber geometries can give rise to two types of errors during network reconstruction: absent connections (false negatives) and spurious connections (false positives; Dauguet et al., 2007; Jbabdi & Johansen-Berg, 2011). These errors cannot be completely eliminated from the reconstructed network; however, when there are multiple subjects, a group threshold can be used to minimize these errors and achieve a balance between the exclusion of false positives and false negatives (de Reus & van den Heuvel, 2013; Roberts, Perry, Roberts, Mitchell, & Breakspear, 2017).

In a recent study, these two types of errors were investigated in undirected connectomes, where false negative connections were generated by pruning existing connections and false positive connections were generated by connecting pairs of unconnected nodes (Zalesky et al., 2016). False positive connections were at least twice as detrimental as false negatives to the estimation of common graph-theoretic measures: clustering coefficient, network efficiency, and modularity. This has been attributed to the modular topology of the network (Sporns & Betzel, 2016). Because nodes within the same module are likely to have a higher connection density, false negative connections were more likely to occur within modules and to be more redundant to network topology. Conversely, false positive connections were more likely to occur between modules, introducing shortcuts that have a greater impact on the graph-theoretic metrics of the network. Here we investigated the impact of perturbations to a subset of unidirectional connections, which were about half intramodular and half intermodular. Despite the similarity of this analysis, here we generated false negative connections by removing existing unidirectional connections and false positive connections by adding the reciprocal connections and making them bidirectional.

Our results also show that false positive connections were overall more detrimental than false negatives. This occurs for betweenness, path length, global efficiency, and participation index. Notably, the small-world index and the clustering (for some connectomes) are exceptions, in which false negative directed connections are more detrimental than false positives. For these measures, the removal of directed connections reduces the number of closed three-node motifs in the network, which may be more detrimental. These findings suggest that graph-theoretic measures are overall more susceptible to addition of shortcuts introduced by false positive connections. A simple and immediate recommendation that follows from our results is that connectomes should be thresholded stringently to maximize specificity at the cost of sensitivity. This recommendation is very straightforward to implement and does not require the development of any new methodologies. In the mouse as well as other connectomes that have weaker unidirectional connections, a more stringent thresholding would create more false negative unidirectional connections and avoid many false positive unidirectional connections that are more detrimental for network measures. Our findings also suggest that the development of future connectome mapping methodologies should place more importance on specificity. In this way, our work can inform and guide the development of future tractography algorithms.

### Connectome Mapping and Directionality Estimation

For the reconstruction of the macroscopic human connectome, parcellation schemes range from less than 10^2^ nodes or regions up to more than 10^5^ (see, for example, Aleman-Gomez, 2006; Glasser et al., 2016; Hagmann et al., 2007; Salvador, Suckling, Schwarzbauer, & Bullmore, 2005; Tzourio-Mazoyer et al., 2002; van den Heuvel, Stam, Boersma, & Pol, 2008). The choice of parcellation can affect several local and global topological parameters of the network, lowering the reliability of comparisons between connectomes (Zalesky et al., 2010). The parcellation also affects the proportion of unidirectional connections, as coarser parcellations correspond to larger brain regions that are more likely to have reciprocal connections. For example, three of the connectomes can be considered coarse parcellations and have a relatively small proportion of unidirectional connections (macaque *N* = 47, *N* = 71, and cat connectomes). Nonetheless, even for these connectomes, the identification of hubs and their graph-theoretic measures can result in inaccuracies due to loss of connection directionality.

We have used connectomes from various species and parcellations that were obtained using different techniques. These factors make it a complex task to compare and interpret some subtle features of the results across all connectomes. Nonetheless, the consistency of most results across connectomes suggests that they reflect general properties of brain networks and are largely independent from the techniques used to obtain these connectomes. Hence, they are also expected to be valid in other connectomes.

### Effect of Connectome Structure on Brain Dynamics

Although the problem of directionality is a recurrent topic in connectomics, with few exceptions (Négyessy, Nepusz, Zalányi, & Bazsó, 2008; Rosen & Louzoun, 2014), most work has focused on identifying the directionality of the interactions from the dynamics of nodes. The directionality of the interactions of nodes in motifs and networks is paramount to shaping the dynamics of systems (Bargmann & Marder, 2013). The dynamics of small circuits or network motifs can be substantially altered by subtle differences in connectivity patterns. For example, the presence of a single reciprocal connection can amplify the synchronization due to resonance (Gollo & Breakspear, 2014; Gollo, Mirasso, Sporns, & Breakspear, 2014); the presence of triangles (loops) can increase metastability (Gollo & Breakspear, 2014) or multistability (Levnajić, 2011) due to frustration. Moreover, the presence of an inhibitory feedback can cause anticipated synchronization between neurons (Matias, Gollo, Carelli, Mirasso, & Copelli, 2016) or cortical regions (Matias et al., 2014). Naturally, this susceptibility of the dynamics to structural perturbations goes beyond network motifs, affecting the dynamics of the whole network (Eguíluz, Pérez, Borge-Holthoefer, & Arenas, 2011; Esfahani, Gollo, & Valizadeh, 2016; Gollo, Zalesky, Hutchison, van den Heuvel, & Breakspear, 2015; Hu, Trousdale, Josić, & Shea-Brown, 2012).

A basic and influential manner of summarizing the dynamics of brain networks corresponds to functional connectivity (Biswal et al., 1995). Functional connections correspond to linear correlations between pairs of regions. These functional connections are symmetric and undirected (Friston, 2011). Disambiguating the directionality of connections between pairs of cortical regions has been a priority in the field (Friston, 2011; Friston, Harrison, & Penny, 2003), as this directionality can reveal causal interaction between regions, or how they effectively interact (Friston et al., 2017). Furthermore, a number of methods have been proposed and utilized to determine the causal interactions between nodes (Friston, Moran, & Seth, 2013), or to reconstruct the underlying network structure from the network dynamics (Ching & Tam, 2017; Deng, Deng, Yu, Guo, & Wang, 2016; Friston et al., 2013; López-Madrona, Matias, Pereda, Canals, & Mirasso, 2017; Napoletani & Sauer, 2008; Stam, Nolte, & Daffertshofer, 2007; Tajima, Yanagawa, Fujii, & Toyoizumi, 2015; Timme, 2007; Vicente, Wibral, Lindner, & Pipa, 2011; Wei, Liao, Yan, He, & Xia, 2017). A better understanding of the relationship between directionality in network structure and dynamics may aid in determining causal interactions (Stephan et al., 2009).

At the network level, it is important to distinguish the roles of in- and out-degree in affecting brain dynamics. A recent study found strong relationships between the structural connectivity of a region and its BOLD (blood oxygen level dependent) signal dynamics (Sethi et al., 2017). Furthermore, several graph-theoretic measures showed stronger correlations to the network dynamics (resting-state functional MRI) when directionality was taken into account. Brain regions receiving more input (larger in-degree) required longer integration time to process and combine all these inputs, which is consistent with the attributed function of rich-club association areas (Heeger, 2017), and also supports the notion of a hierarchy of timescales recapitulating the anatomical hierarchy of brain structure (Chaudhuri, Knoblauch, Gariel, Kennedy, & Wang, 2015; Cocchi et al., 2016; Gollo, Roberts, & Cocchi, 2016; Gollo et al., 2015; Kiebel, Daunizeau, & Friston, 2008; Murray et al., 2014). Overall, these findings highlight the importance of the directionality of the structural connectivity to understand brain dynamics.

Despite intensive efforts, the structure-function relationship remains far from elucidated, and the issue of inferring directionality in undirected anatomical connectomes has yet to be addressed. Here we have focused on characterizing the effect of directionality on brain structure via graph-theoretic measures, and future work will characterize how perturbations to the directionality of connections influence network dynamics.

## CONCLUSIONS

Connectomes are inherently directed networks. The majority of noninvasive techniques for mapping connectomes are unable to resolve connection directionality, thereby yielding undirected approximations in which truly unidirectional connections are either overlooked or rendered bidirectional. We found that the inability to resolve connection directionality can introduce substantial error to the estimation of topological descriptors of brain networks, particularly with respect to the classification and identification of hubs. We analyzed the effect of progressively eliminating connection directionality information in six directed connectomes that were mapped with invasive techniques capable of resolving afferent and efferent connections (*C. elegans*, mouse, cat, and three macaque networks). We demonstrated that the identification of the most connected hubs is especially affected by the loss of connection directionality. We also found that the addition of reciprocal unidirectional connections (false positives) is more detrimental to the estimation of most topological measures than removal of unidirectional connections (false negatives). Our findings underscore the need for noninvasive connectome mapping techniques that can (a) provide estimates of connection directionality and (b) yield relatively sparse and highly specific fiber maps that preference false negatives over false positives. Given that most topological properties have been found to be recapitulated across directed (macaque) and undirected (human) connectomes, at least qualitatively, resolving the directionality of human connectomes in the future will most likely not result in a radical reappraisal of human brain network organization, but it will enable a more accurate characterization of the human connectome.

## ACKNOWLEDGMENTS

We would like to sincerely thank Madeleine Flynn, QIMR Berghofer Medical Research Institute, for her illustrations (Figure 1 brain/nervous system images).

## AUTHOR CONTRIBUTIONS

Penelope Kale: Formal analysis; Investigation; Resources; Visualization; Writing – original draft; Writing – review & editing. Andrew Zalesky: Conceptualization; Validation; Writing – review & editing. Leonardo L. Gollo: Conceptualization; Funding acquisition; Methodology; Project administration; Resources; Supervision; Validation; Writing – original draft; Writing – review & editing.

## FUNDING INFORMATION

Leonardo L. Gollo, National Health and Medical Research Council (http://dx.doi.org/10.13039/501100000925), Award ID: APP1110975. Andrew Zalesky, National Health and Medical Research Council (http://dx.doi.org/10.13039/501100000925), Award ID: APP1047648.

## TECHNICAL TERMS

Undirected network: A network describing the presence (or the strength) of a relationship between nodes for which the edges are represented in the absence of information about the directionality of the connection.
Graph theory: A branch of mathematics concerned with the study of networks (graphs). It provides various quantitative measures that are used to describe the topological organization of networks.
Unidirectional connection: An edge in a directed graph denoting a connection from one node to another in a single direction.
False positive connection: A connection that is spuriously represented in a connectivity matrix without the existence of an underlying link. False positive connections often occur because of errors associated with the inference of connections. In undirected networks, we use the expression false positive connection to refer to a unidirectional connection that is represented as an undirected link, which is often assumed to be a bidirectional connection.
False negative connection: A connection that is not represented in a connectivity matrix given the existence of an underlying link. False negative connections often occur because of errors associated with the inference of connections. In undirected networks, we use the expression false negative connection to refer to a unidirectional connection that is not represented as an undirected link.
Bidirectional connection: An edge in a directed graph denoting a reciprocal connection between two nodes.
Structural connectivity: A description of the presence (and potentially directionality and weight) of anatomical connections (e.g., synapses or axonal tracts) between (brain network) nodes such as cortical areas or neurons.
Parcellation: A segmentation of the brain into individually defined parcels; these parcels often refer to cortical areas, the nodes of macroscale connectomes.
Connector hubs: A highly connected hub region that has a substantial density of connections with regions from other modules.
Provincial hubs: A highly connected hub region that has a substantial density of connections with regions belonging to the same module.
