# Supplementary material for: Estimating the impact of structural directionality: How reliable are undirected connectomes?
Source: Netw Neurosci. 2018 Jun 1;2(2):259–84. doi: 10.1162/netn_a_00040 (PMC6135560; doi:10.1162/netn_a_00040)
Supplement: Supplementary file 1 [file netn-02-259-s001.pdf]

Kale, P., Zalesky, A., & Gollo, L.L. (2018). Supplementary Material for "Estimating the impact of structural directionality: How reliable are undirected connectomes?" *Network Neuroscience, 2*(2), 259-284.
https://doi.org/10.1162/netn_a_00040

Supplementary Material

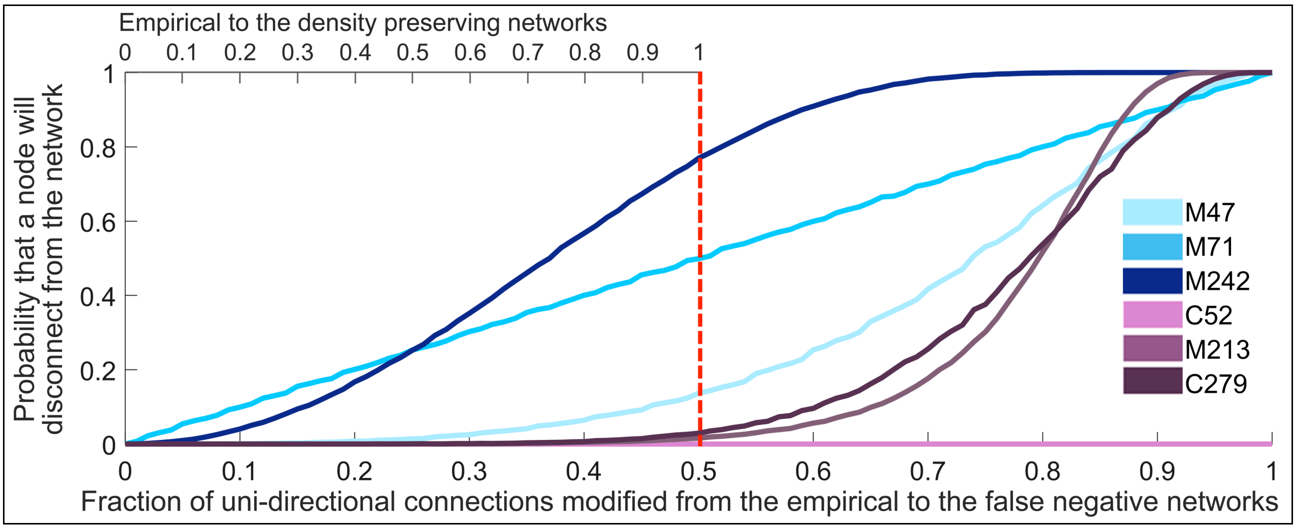

Supplementary Figure 1: **Probability that a node will disconnect from the density preserving and false negative networks.** Probability that a node will disconnect from the network as uni-directional connections are removed in the false negative perturbed networks (lower x-axis). The probability of nodal disconnection for the density preserving networks (top x-axis prior to the red dashed line) as randomly selected uni-directional connections are removed, with a reciprocal connection added to another uni-directional connection with each modification. These results describe the mean over 1000 trials. M47: the macaque connectome with 47 nodes, M71: macaque N=71, M242: macaque N=242, C52: cat, M213: mouse, C279: C. elegans.

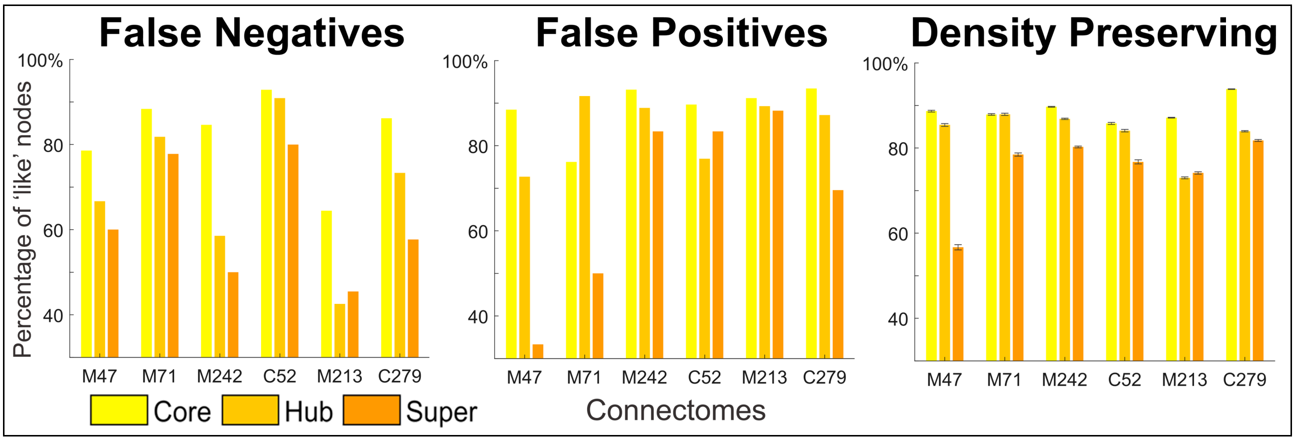

Supplementary Figure 2: **Percentage of high degree nodes classified correctly as defined by the empirical network.** These figures display the percentage of core, hub, and super-hub nodes across all connectomes that are accurate (see methods) according to those in the empirical networks when these nodes are redefined for each of the perturbed connectomes (with 100% of uni-directional connections altered; the density-preserving results show a mean over 1000 trials with the standard deviation). M47: the macaque connectome with 47 nodes, M71: macaque N=71, M242: macaque N=242, C52: cat, M213: mouse, C279: C. elegans.

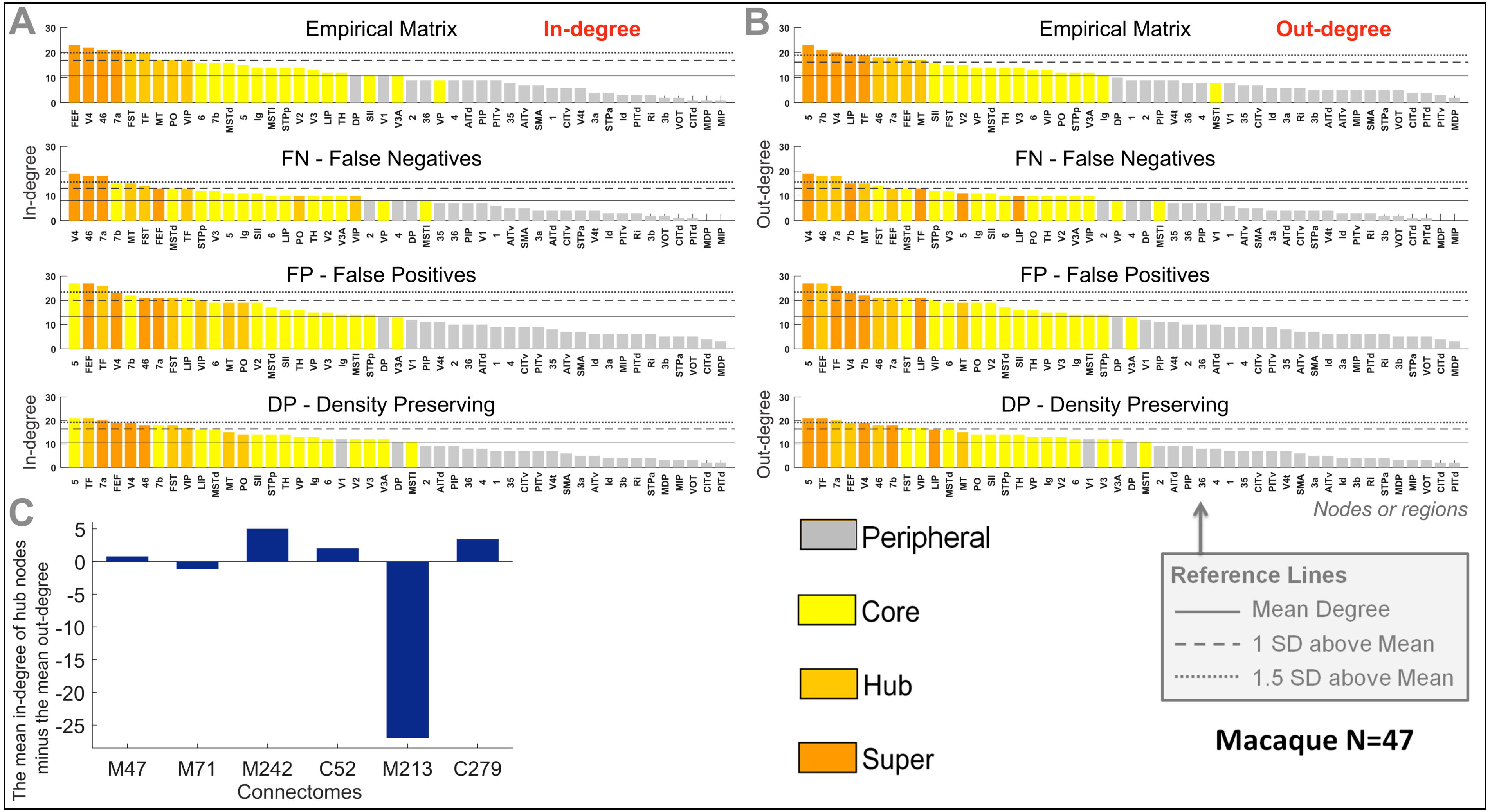

Supplementary Figure 3: **Directionality effects on the in- and out- degree.** (A-B) Cortical areas of the macaque N=47 connectome sorted by in-degree (A) or out-degree (B) for the empirical and each perturbed network. Hubs are defined as nodes that have a total in-degree (A) or out-degree (B) one standard deviation above the mean, and super-hubs are defined as nodes that have an in-/out-degree 1.5 standard deviations

above the mean (the density-preserving results are from an illustrative single trial). (C) The mean of the in-degree minus the out-degree for the set of hub nodes in each connectome. M47: the macaque connectome with 47 nodes, M71: macaque N=71, M242: macaque N=242, C52: cat, M213: mouse, C279: C. elegans.

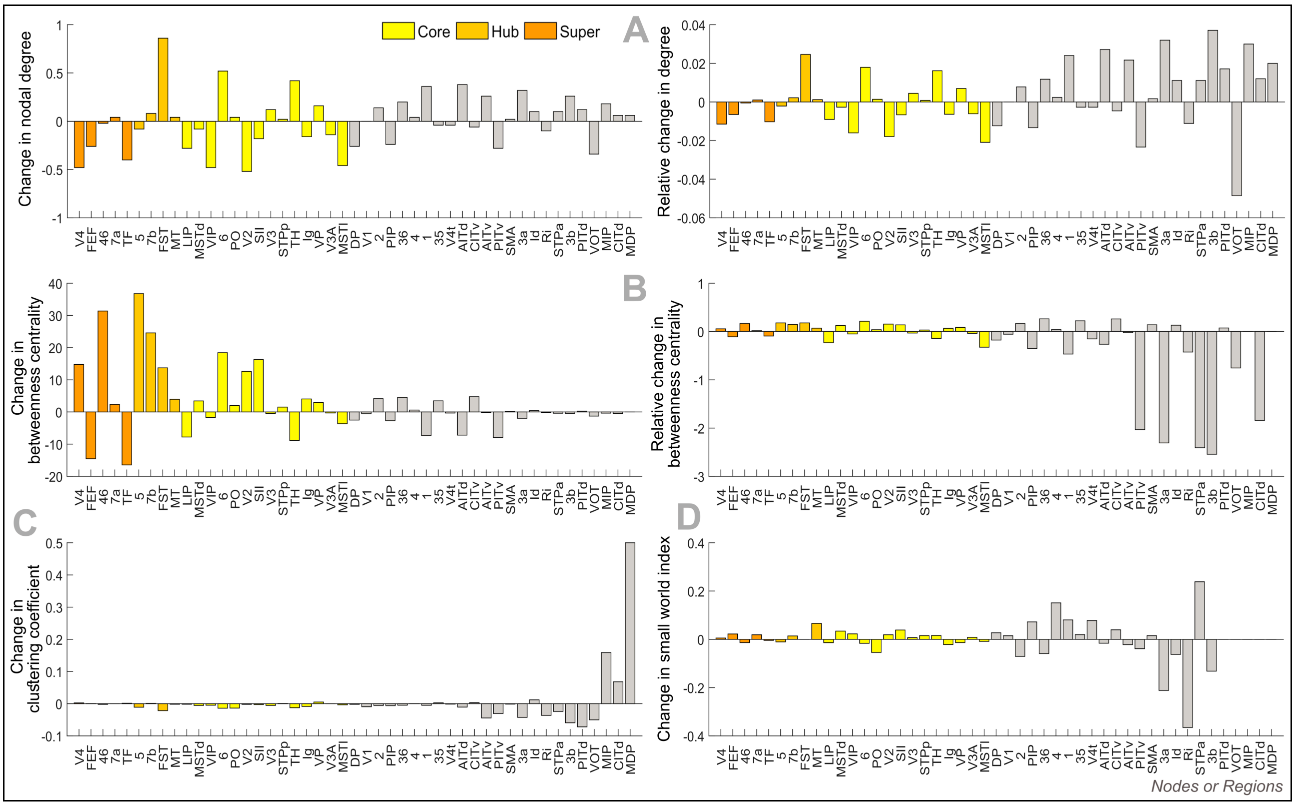

Supplementary Figure 4: **Nodal variation in graph-theoretic measures for each region in the macaque N=47connectome from the empirical to the density preserving network.** (A) Change in the degree of each region (left) from the empirical to the density preserving network, and the relative change (right). (B) Change in the betweenness centrality of each node (left) and the relative change (right). (C) Change in the clustering coefficient of each node. (D) The change in the small-world index of each node ($S_i^{\rightarrow}$). These results were generated from the mean change across 50 trial density preserving networks, and were relative to the maximum graph-theoretic value (for each measure and region) across the macaque empirical network.

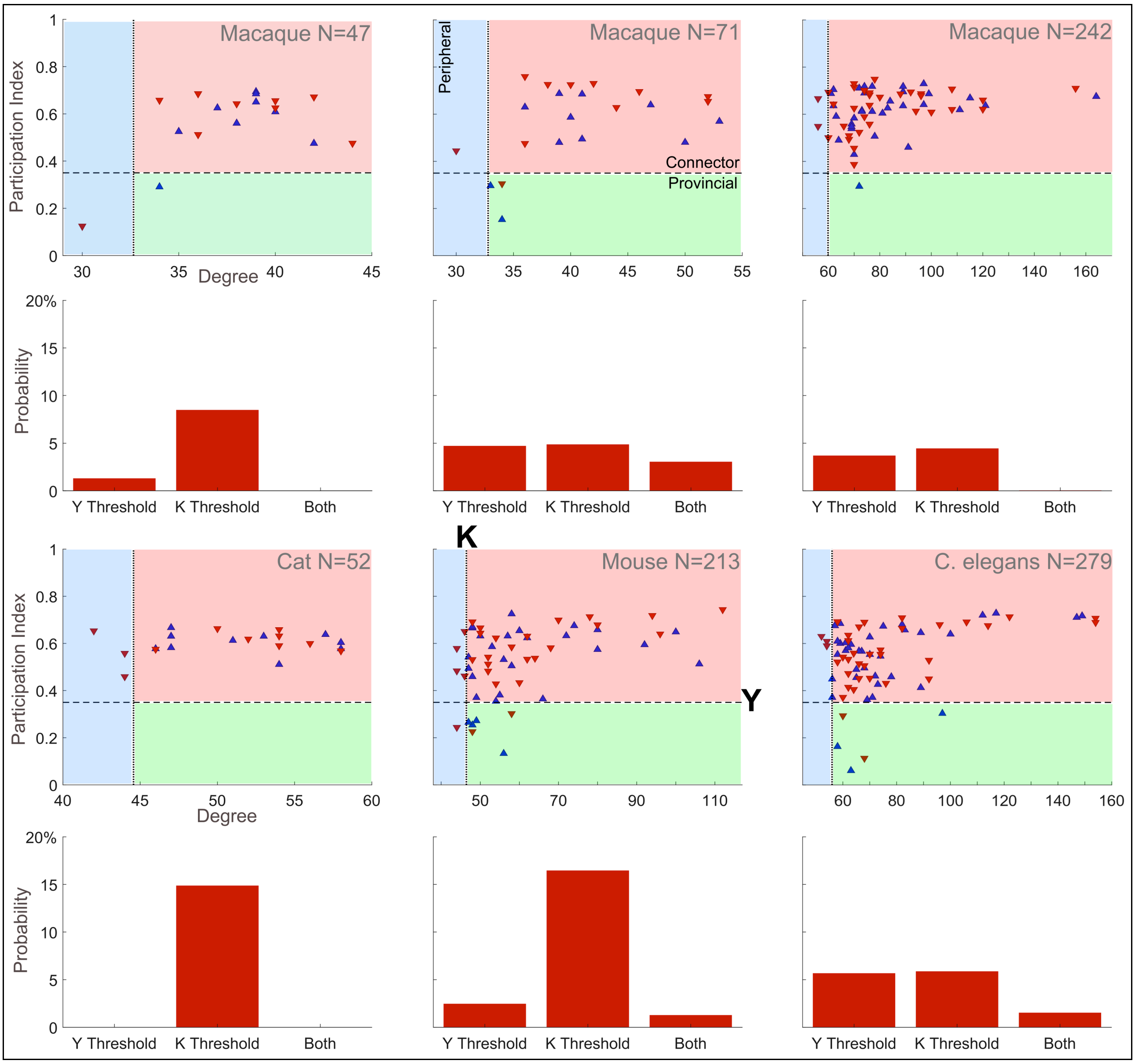

Supplementary Figure 5: **Relationship between the participation index and degree for hub nodes in each empirical and density preserving connectome.** Plots (participation vs. degree) displaying the hub nodes of the empirical (blue) network, and the same regions in the density preserving (red) networks for each connectome (taken from a single illustrative trial). The dotted line represents the hub definition based on the degree (K > one standard deviation above the mean), and the dashed line represents the further classification of hubs based on the participation index (connector hubs Y > 0.35 and provincial hubs Y ≤ 0.35). The bar figures below show the probability that a hub node will cross over either, or both of the threshold lines following density-preserving alterations in directionality (over 1000 trials), resulting in a classification that is inconsistent with the empirical connectome.

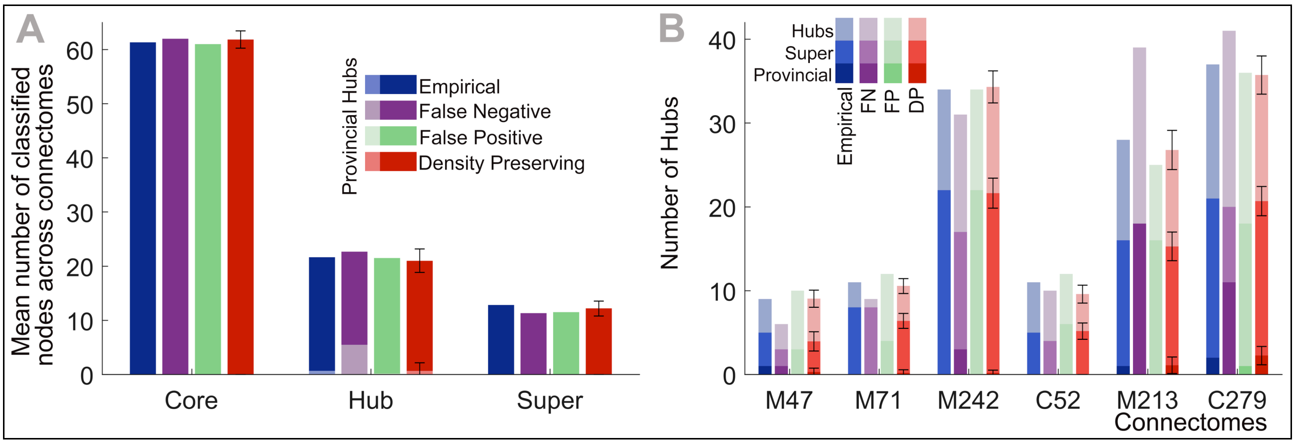

Supplementary Figure 6: **Number of core, hub and super-hub nodes identified in each empirical and perturbed network across all connectomes.** (A) Mean number of each type of highly connected region across all connectomes, for the empirical and each perturbed network (100% of uni-directional connections altered). (B) Number of each type of hub region across all connectomes, for the empirical and each perturbed network. Results for the density preserving network are the mean of 50 trials and show the standard deviation. M47: the macaque connectome with 47 nodes, M71: macaque N=71, M242: macaque N=242, C52: cat, M213: mouse, C279: C. elegans.

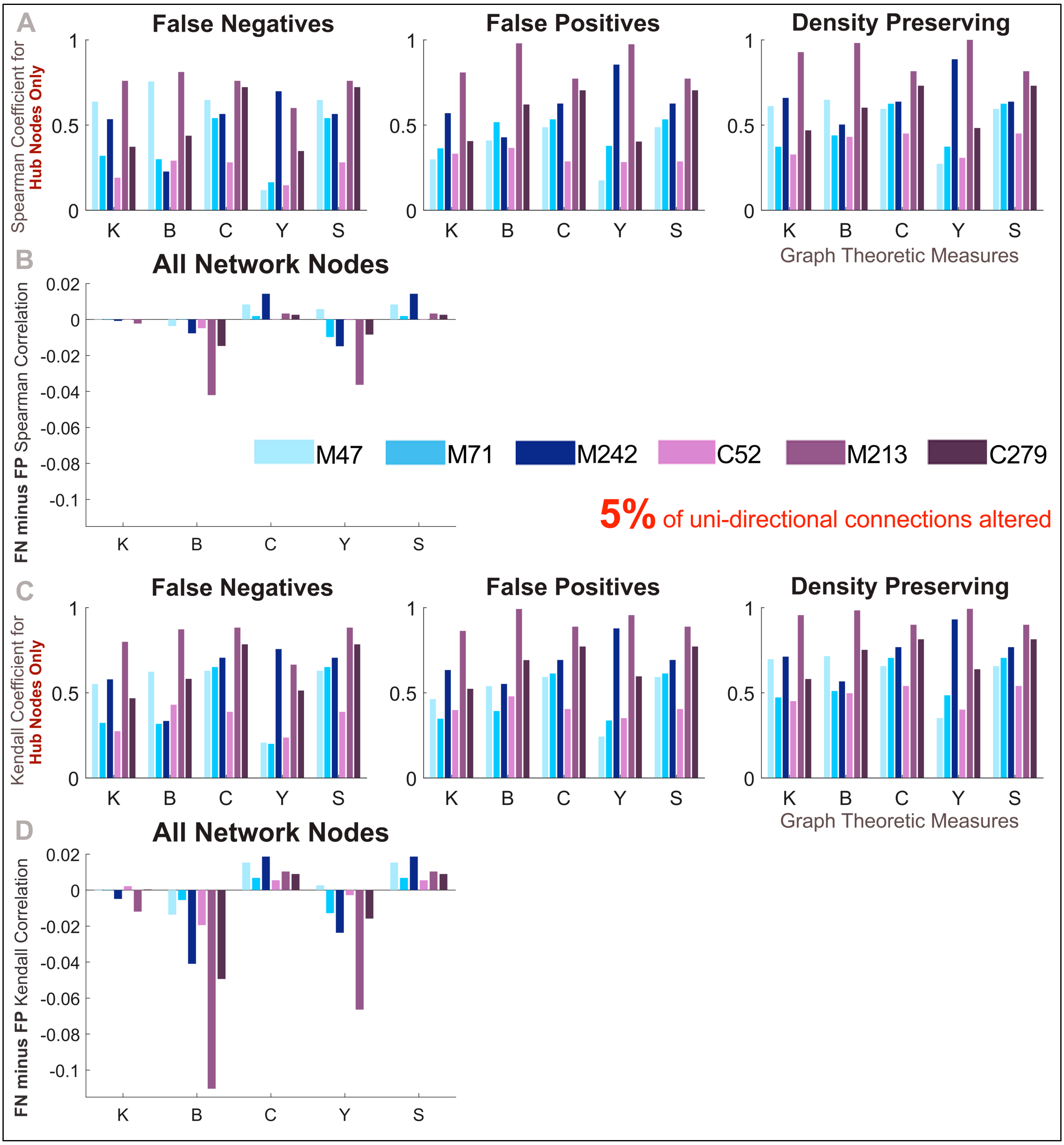

Supplementary Figure 7: **Nodal changes measured by the Spearman correlation and Kendall coefficient.** (A) Spearman correlation of hub nodes across all perturbed networks, for each graph-theoretic measure. (B) Difference in the spearman correlation between the false-negative and false-positive networks for all nodes in the network. A positive value indicates the false-negative connections cause greater changes in the ranking of nodes, whereas a negative value indicates the same for false-positive connections. (C) Kendall coefficient of hub nodes across all perturbed networks, for each graph-theoretic measure. (D) Difference in the Kendall

coefficient between the false-negative and false-positive networks for all nodes in the network. (A-D) Results correspond to the mean over 50 trials for which 5% of randomly selected uni-directional connections are modified in each perturbed network. Graph-theoretic measures are as follows: K=Degree, B=Betweenness centrality, C=Clustering coefficient, Y=Participation index and S=Small-world index ($S_i^{\rightarrow}$). M47: the macaque connectome with 47 nodes, M71: macaque N=71, M242: macaque N=242, C52: cat, M213: mouse, C279: C. elegans.

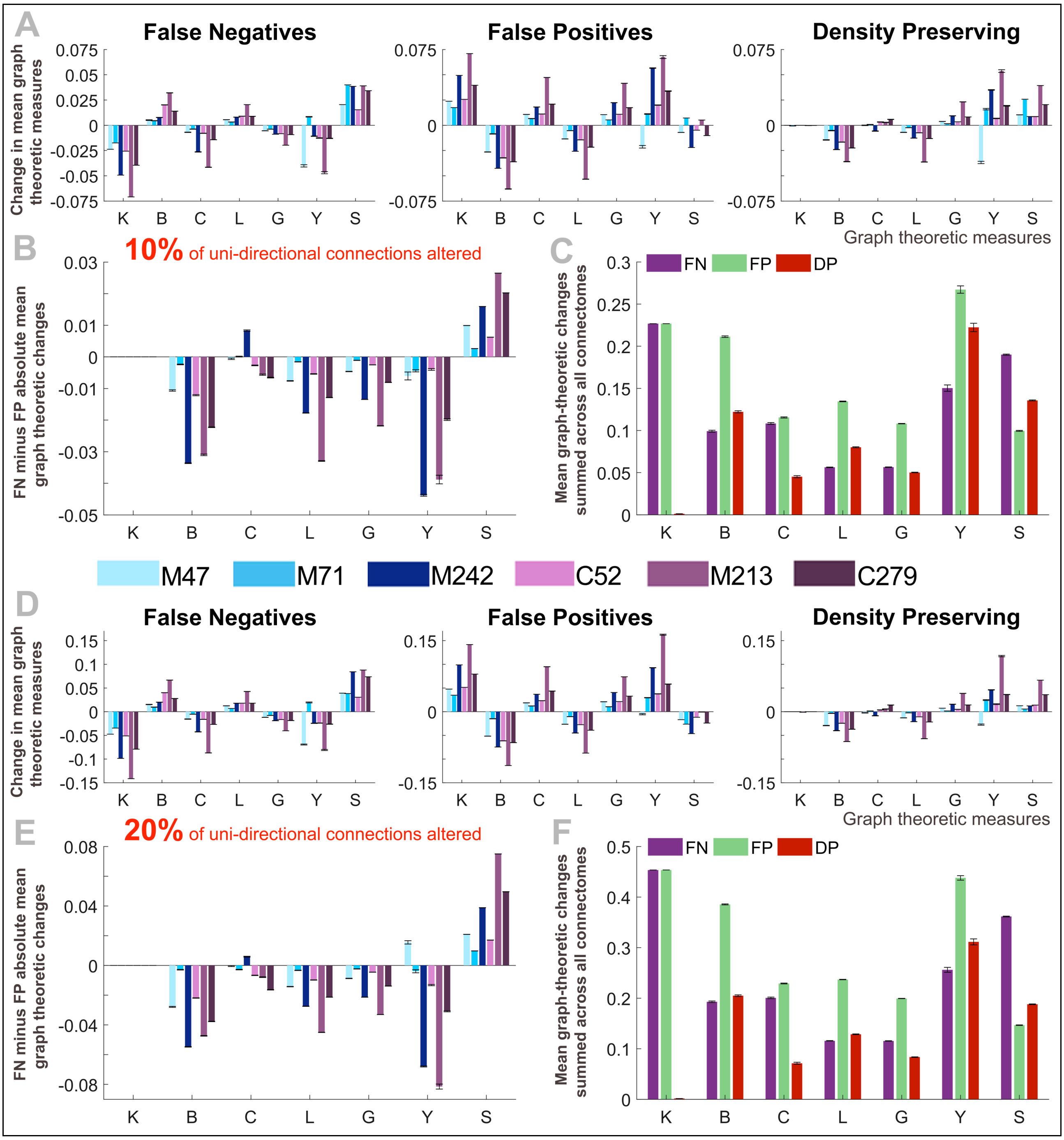

Supplementary Figure 8: **Relative change in mean graph-theoretic measures from the empirical connectomes for perturbed networks with 10% and 20% of uni-directional connections altered.** Changes in mean graph-theoretic measures across all connectomes and each type of perturbed network with 10% of uni-directional connections altered (A) and 20% of uni-directional connections altered (D). Difference between the changes in mean graph-theoretic measures for the 10% (B) and 20% (E) false-negative and false-positive networks. Mean changes in graph-theoretic measures for each of the perturbed networks with 10% of connections altered (C) or 20% of connections altered (F) summed across all connectomes. (A-

F) The results represent the mean of these networks over 50 trials, and describe the change in the mean graph-theoretic measure (from the empirical to perturbed network) normalized by the mean of the empirical network (error bars show the standard error of the mean). Graph-theoretic measures are as follows: K=Degree, B=Betweenness centrality, C=Clustering coefficient, L=Characteristic path length, G=Global efficiency, Y=Participation index and S=Small-world index ($S^{\rightarrow}$, this measure is the mean over 1000 trials). M47: the macaque connectome with 47 nodes, M71: macaque N=71, M242: macaque N=242, C52: cat, M213: mouse, C279: C. elegans.

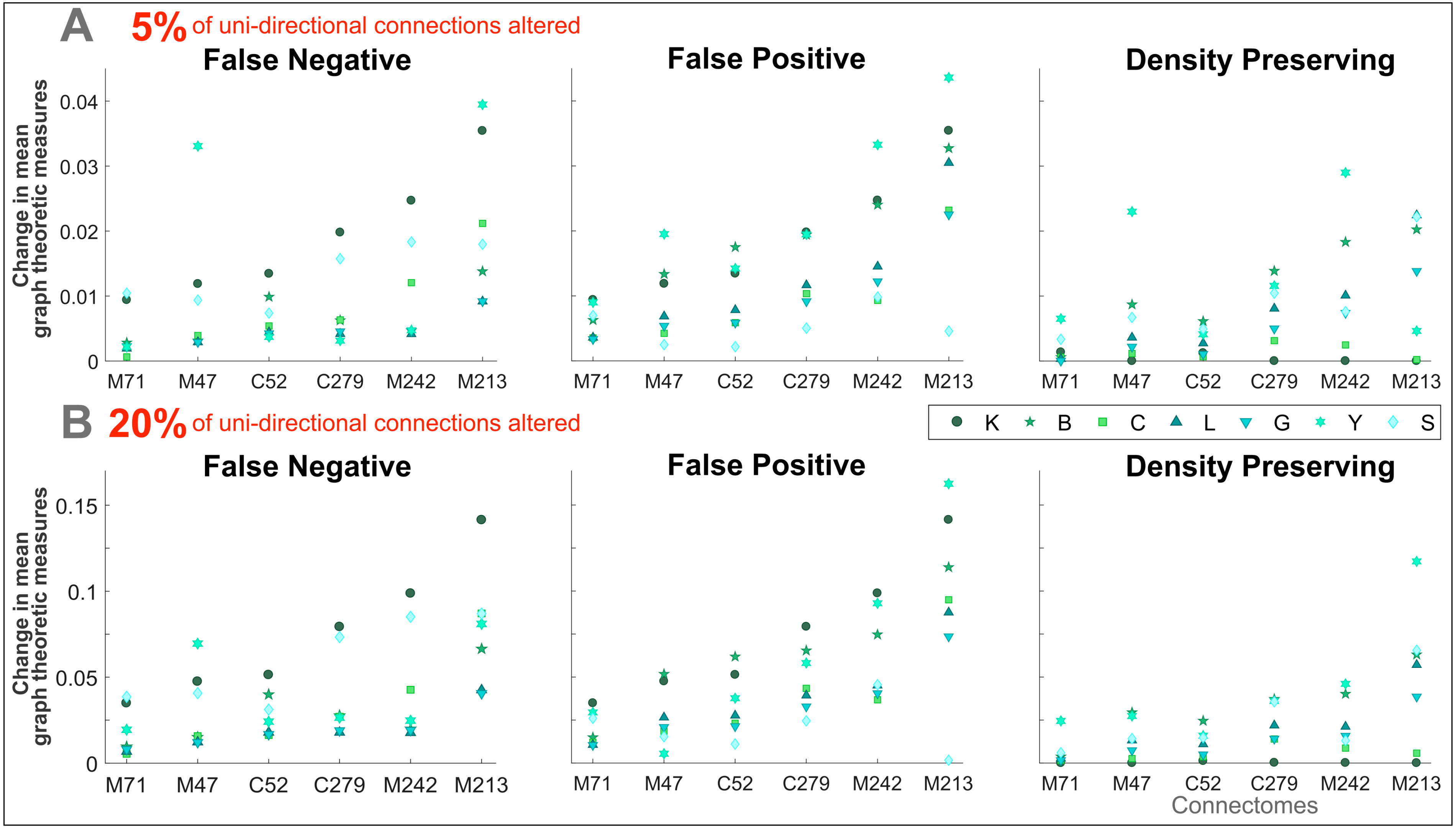

Supplementary Figure 9: **The change in mean graph-theoretic measures across connectomes sorted by the proportion of uni-directional connections (M71 = 17%, M47 = 24%, C52 = 26%, C279 = 40%, M242 = 49%, M213 = 71%).** (A) Relative change in mean graph theoretic measures (see legend) for each type of perturbed network with 5% of uni-directional connections altered. (B) Results for perturbed networks

with 20% of uni-directional connections altered. The results represent the mean of these networks over 50 trials, and describe the change in the mean graph-theoretic measure (from the empirical to perturbed network) normalized by the mean of the empirical network. The legend shows each graph theoretic measure as follows: K=Degree, B=Betweenness centrality, C=Clustering coefficient, L=Characteristic path length, G=Global efficiency, Y=Participation index and S=Small-world index ($S^{\rightarrow}$, this measure is the mean over 1000 trials). M47: the macaque connectome with 47 nodes, M71: macaque N=71, M242: macaque N=242, C52: cat, M213: mouse, C279: C. elegans.

Connectome		Macaque	Macaque	Macaque	Cat	Mouse	C. elegans
**Number of**	Nodes	47	71	242	52	213	279
	Modules	4	4	5	3	5	4
	Connections	505	746	4090	818	3301	4903
**Proportion of**	Uni-directional connections	0.24	0.17	0.49	0.26	0.71	0.40
**Density of**	All connections	0.23	0.15	0.07	0.31	0.07	0.06
	Uni-directional connections	0.06	0.03	0.03	0.08	0.05	0.03
**Number of connections**	Inter-/intra-modular ratio	0.51	0.39	0.63	0.53	0.58	0.45
	Core	263	337	2080	362	1025	1763
	Feeder	194	339	1705	346	1729	2117
	Peripheral	48	70	305	110	547	1023
**Proportion of uni-directional connections**	Core	0.19	0.12	0.43	0.15	0.63	0.37
	Feeder	0.29	0.21	0.56	0.39	0.76	0.43
	Peripheral	0.29	0.29	0.59	0.20	0.70	0.37
**Density of**	Hub-hub connections	0.69	0.57	0.43	0.85	0.27	0.35
	Hub-peripheral connections	1.64	1.55	0.75	1.62	1.37	0.66
**Proportion of uni-directional connections**	Hub-hub connections	0.16	0.05	0.30	0.05	0.54	0.31
	Hub-peripheral connections	0.20	0.19	0.43	0.26	0.84	0.36
**Mean**	Degree	21.5	21.0	33.8	31.5	31.0	35.1
	Betweenness centrality	48	92	368	41	335	417
	Clustering coefficient	0.58	0.47	0.37	0.59	0.28	0.29
	Participation index	0.378	0.288	0.380	0.381	0.325	0.332
	Small-world index	2.23	2.78	4.71	1.79	3.31	4.20
**Network measures**	Characteristic path length	2.05	2.33	2.53	1.81	2.63	2.50
	Global efficiency	0.57	0.50	0.44	0.64	0.43	0.44

Supplementary Table 1: **Network characteristics of each empirical connectome.** Details of modularity determination are presented in Methods.

	Proportion of randomly selected connections	Modularity in perturbed networks	Number of trials	Connectomes	Definition of hubs in perturbed networks
Fig. 1	-	-	-	All	-
Fig. 2	100%	Redefined	1	M47	-
Fig. 3	100%	Redefined	**A**: 1, **B**: 1	All	-
Fig. 4	100%	Redefined	**A**, **C&D**: 1, **B&E**: 1000	**A**, **C&D**: M47, **B&E**: All	**A&C-E**: Empirical, **B**: Redefined
Fig. 5	5%	Empirical	50	All	Empirical
Fig. 6	5%	Empirical & Redefined	50, except *small world index*: 1000	All	-
S.Fig. 1	0-100%	-	10, 000	All	-
S.Fig. 2	100%	-	1	**A**, **B**: M47, **C**: All	**A**, **B**: As per *in-/ out- degree* of Empirical network, **C**: Empirical
S.Fig. 3	100%	-	1000	All	Redefined
S.Fig. 4	100%	-	50	M47	Empirical
S.Fig. 5	100%	Redefined	Plots: 1, Bars: 1000	All	Empirical
S.Fig. 6	100%	Both Displayed	50	All	Redefined
S.Fig. 7	5%	Empirical	50	All	Empirical
S.Fig. 8	10% and 20%	Redefined	50, except *small world index*: 1000	All	-
S.Fig. 9	5% and 20%	Redefined	50, except *small world index*: 1000	All	-

Supplementary Table 2: **Methodological details for analyses presented in each figure and in the supplementary material.** M47: the macaque connectome with 47 nodes.

Graph-theoretic Measure		Formula
**Degree** (Rubinov and Sporns, 2010)	K_i= degree of node i (sum of directed in- and out-degree)	(both) $K_i = \sum_{j \in N} a_{ij}$ $K_i^{in} = \sum_{j \in N} a_{ji}$ $K_i^{out} = \sum_{j \in N} a_{ij}$
**Betweenness Centrality** (Freeman, 1978)	B_i= betweenness centrality of node i, $B_{hj}(i)$ = number of shortest paths between h &j passing through i,B_{hj}= number of shortest paths between h &j	$B_i = \frac{1}{(n-1)(n-2)} \sum_{h,j \in N, h \neq j, h \neq i, j \neq i} \frac{B_{hj}(i)}{B_{hj}}$
**Number of Triangles** (Rubinov and Sporns, 2010)	$t_i^{\rightarrow}$= number of triangles around node i $t_i^{\rightarrow} = \frac{1}{2} \sum_{j,h \in N} (a_{ij} + a_{ji})(a_{ih} + a_{hi})(a_{jh} + a_{hj})$	
**Clustering Coefficient** (Fagiolo, 2007)	$C_i^{\rightarrow}$= clustering coefficient of node i $C_i^{\rightarrow} = \frac{1}{n} \sum_{i \in N} \frac{t_i^{\rightarrow}}{(K_i^{out} + K_i^{in})(K_i^{out} + K_i^{in} - 1) - 2 \sum_{i \in N} a_{ij} a_{ji}}$ $C^{\rightarrow}$= mean clustering coefficient of the network $C^{\rightarrow} = \sum_{i=1}^{N} \frac{C_i^{\rightarrow}}{N}$	
**Shortest Path Length** (Rubinov and Sporns, 2010)	$d_{ij}^{\rightarrow}$ = shortest path length between nodes i & j, where $g_{i \rightarrow j}$ is the shortest path between i & j	$d_{ij}^{\rightarrow} = \sum_{a_{ij} \in g_{i \rightarrow j}} a_{ij}$
**Characteristic Path Length** (Watts and Strogatz, 1998)	$L^{\rightarrow}$ = average distance between all nodes	$L^{\rightarrow} = \frac{1}{n} \sum_{i \in N} \frac{\sum_{j \in N, j \neq i} d_{ij}^{\rightarrow}}{n-1}$
**Global Efficiency** (Latora and Marchiori, 2001)	$G^{\rightarrow}$ = global efficiency of the network	$G^{\rightarrow} = \frac{1}{n} \sum_{i \in N} \frac{\sum_{j \in N, j \neq i} (d_{ij}^{\rightarrow})^{-1}}{n-1}$
**Participation Index** (Guimera and Amaral, 2005)	Y_i^{out}=out-participation index, M= set of modules, $K_i^{out}(m)$ = number of out-connections between i & all nodes in module m	$Y_i^{out} = 1 - \sum_{m \in M} \left(\frac{K_i^{out}(m)}{K_i^{out}} \right)^2$
**Small Worldness** (Humphries and Gurney, 2008)	$S_i^{\rightarrow}$ = small worldness of node i $S^{\rightarrow}$ = small world index of network $C_{i\,rand}^{\rightarrow}$ = clustering of a random network, $L_{rand}^{\rightarrow}$ = path length of a random network	$S_i^{\rightarrow} = \frac{C_i^{\rightarrow} / C_{i\,rand}^{\rightarrow}}{L^{\rightarrow} / L_{rand}^{\rightarrow}}$ $S^{\rightarrow} = \frac{C^{\rightarrow} / C_{rand}^{\rightarrow}}{L^{\rightarrow} / L_{rand}^{\rightarrow}}$

Notations: N = all nodes in the network, n = number of nodes, L = all connections, l = number of connections, (i, j) = connection between nodes i & j, (i, $j \in N$), a_{ij} = connection status between i & j: = 1 when a connection from node i to j exists, otherwise = 0, $l = \sum_{i,j \in N} a_{ij}$(each bidirectional connection is counted twice, as a_{ij} & as a_{ji}), *rand* = random network, → indicates formulae that consider directionality

Supplementary Table 3: **Reference, description and formula for each graph-theoretic measure used in this study.** Table adapted from Rubinov and Sporns (2010).

	M47	M71		M242				C52		M213			C279			
**Connector**	V4	A46		46	7b	23	25	CGp	EPp	GPi	LGv	MH	AVAR	PVPL	AIBL	RMGL
	FEF	TF		24	8A	S2	46v	35		STN	VM	MPT	AVAL	AVDL	RIAL	AVG
	46	TPT		LIP	23c	24c	24b	AES		CLA	RM	PA	AVBR	PVT	RIBL	DVC
	7a	A7a		32	PIT	PS		36		CLI	MM	PTLp	AVBL	AVHR	RIBR	HSNR
	TF	V4		TF	10	6M		Ia		PP	NOT	SPFm	DVA	PVR	AVJR	RIMR
	5	FEF		13a	TE	ENT		Ig		PPN	MGd	BLA	PVCR	PVNR	RMDL	RIH
	FST	TS3		12o	PGm	8B		7		PERI	RCH	VISam	PVCL	AIBR	AVKL	AVL
	MT	ER		9	13	F5		6m		PT	ILA		AVER	PVPR	AVJL	
		TH		TH	36	14		5Al		ACAd	SUBd		AVDR	AVHL	RIML	
				11	F7	lai		20a		SPFp	LGd		AVEL	RIAR	RIGL	
**Provincial**	7b	A7b	LIP	12l						LGd	PAA		RIML	RIH		

Supplementary Table 4: **Connector and provincial hubs identified for each connectome.** The hub definition based on the degree is K greater than one standard deviation above the mean and further classification of hubs based on the participation index for connector hubs is Y > 0.35 and provincial hubs is Y ≤ 0.35. Each section (and column) is sorted by the highest degree. M47: the macaque connectome with 47 nodes, M71: macaque N=71, M242: macaque N=242, C52: cat, M213: mouse, C279: C. elegans.
